# PD-L1 mediates lung fibroblast to myofibroblast transition through Smad3 and β-catenin signaling pathways

**DOI:** 10.1038/s41598-022-07044-3

**Published:** 2022-02-23

**Authors:** Xia Guo, Christudas Sunil, Oluwaseun Adeyanju, Andrew Parker, Steven Huang, Mitsuo Ikebe, Torry A. Tucker, Steven Idell, Guoqing Qian

**Affiliations:** 1 Department of Cellular and Molecular Biology, The University of Texas Health Science Center at Tyler, 11937 US Highway 271, Tyler, TX 75708 USA; 2 Department of Internal Medicine, Division of Pulmonary and Critical Care Medicine at the University of Michigan, Ann Arbor, USA; 3 The Texas Lung Injury Institute, Tyler, TX USA

**Keywords:** Physiology, Pathogenesis

## Abstract

Programmed death ligand-1 (PD-L1) is an immune checkpoint protein that has been linked with idiopathic pulmonary fibrosis (IPF) and fibroblast to myofibroblast transition (FMT). However, it remains largely unclear how PD-L1 mediates this process. We found significantly increased PD-L1 in the lungs of idiopathic pulmonary fibrosis patients and mice with pulmonary fibrosis induced by bleomycin and TGF-β. In primary human lung fibroblasts (HLFs), TGF-β induced PD-L1 expression that is dependent on both Smad3 and p38 pathways. PD-L1 knockdown using siRNA significantly attenuated TGF-β-induced expression of myofibroblast markers α-SMA, collagen-1, and fibronectin in normal and IPF HLFs. Further, we found that PD-L1 interacts with Smad3, and TGF-β induces their interaction. Interestingly, PD-L1 knockdown reduced α-SMA reporter activity induced by TGF-β in HLFs, suggesting that PD-L1 might act as a co-factor of Smad3 to promote target gene expression. TGF-β treatment also phosphorylates GSK3β and upregulates β-catenin protein levels. Inhibiting β-catenin signaling with the pharmaceutical inhibitor ICG001 significantly attenuated TGF-β-induced FMT. PD-L1 knockdown also attenuated TGF-β-induced GSK3β phosphorylation/inhibition and β-catenin upregulation, implicating GSK3β/β-catenin signaling in PD-L1-mediated FMT. Collectively, our findings demonstrate that fibroblast PD-L1 may promote pulmonary fibrosis through both Smad3 and β-catenin signaling and may represent a novel interventional target for IPF.

## Introduction

Pulmonary fibrosis (PF) is a chronic and progressive interstitial lung condition that is characterized by excessive deposition of extracellular matrix and organization. Idiopathic pulmonary fibrosis (IPF) is the most common form of PF and affects approximately 3 million people worldwide^1–3^. The prognosis of IPF patients remains poor with a median survival of 2 to 3 years after diagnosis^4^, largely due to limited treatment options and lack of effective targets.

Immune checkpoint inhibitors have been proven successful therapeutics in several cancer types including non-small cell lung carcinoma^5,6^. Given the increased recognition of the similarities between IPF and lung cancer^7^, the therapeutic targeting of immune checkpoint proteins in IPF patients is under active investigation^8^. Programmed death ligand-1 (PD-L1) is an immune checkpoint protein that is expressed in epithelial and stromal cells enabling immune tolerance and preventing autoimmune response. It has been linked to tumor progression and resistance to targeted therapies through both immune-related (via interacting with its receptor programmed death protein 1, PD-1) and non-related (tumor intrinsic signaling) mechanisms^9–11^. The role of PD-L1 in pulmonary fibrosis development has likewise been investigated in varying contexts. For example, upregulation of PD-1 on CD4^+^ T cells has been reported to promote pulmonary fibrosis in vivo^12^. Further, blockade of PD-1 or PD-L1 using neutralizing antibodies alleviated bleomycin induced lung injury and collagen deposition^12,13^. These studies identify an important role of PD-L1 as an immune checkpoint in the development of pulmonary fibrosis.

Activation and prolonged survival of myofibroblasts is likewise critical for the development of IPF^14^. The origin of myofibroblasts in fibrotic lung tissues is complex^15^, involving resident fibroblasts, epithelial cells, endothelial cells, fibrocytes, pericytes, and macrophages. In these cell types, fibroblast to myofibroblast transition (FMT) is considered a crucial source of myofibroblasts and inhibition of FMT has been repeatedly shown to reduce or block the progression of pulmonary fibrosis^16–18^. The expression of PD-L1 in lung fibroblasts have been reported in several studies^13,19,20^. Recently, Geng et al. demonstrated that higher PD-L1 expression in lung fibroblasts is associated with increased invasion and migration that drives PF progression in a humanized mouse model of IPF^20^. Consequently, PD-L1 neutralization or knockout dramatically attenuated bleomycin induced pulmonary fibrosis in vivo^20^. In established human and murine lung fibroblast cell lines, knockdown of PD-L1 mitigates TGF-β-induced extracellular matrix production^19^. However, little is known about the mechanisms by which PD-L1 modulates FMT to promote pulmonary fibrosis. PD-L1 has been reported to enter the nuclei of cancer cells, which contributes to the anti-tumor response to PD-1 blockade^21^. A recent study also shows that PD-L1 promotes lung cancer cell growth through activation of β-catenin signaling^22^, the latter has been implicated in pulmonary fibrosis^23^. It remains elusive whether PD-L1 might affect Smad3 and β-catenin signaling in the context of FMT and thus the focus of the present study.

In this study, we report previously unrecognized mechanisms by which intrinsic PD-L1 mediates FMT induced by TGF-β. We found that PD-L1 mediates FMT through interaction with Smad3 and activation of β-catenin signaling. These novel findings provide critical insight on the role of PD-L1 in the development of PF and further support targeting PD-L1 as a potential therapeutic option for IPF.

## Results

### PD-L1 level is upregulated in the lungs of human IPF patients

**Figure 1:**
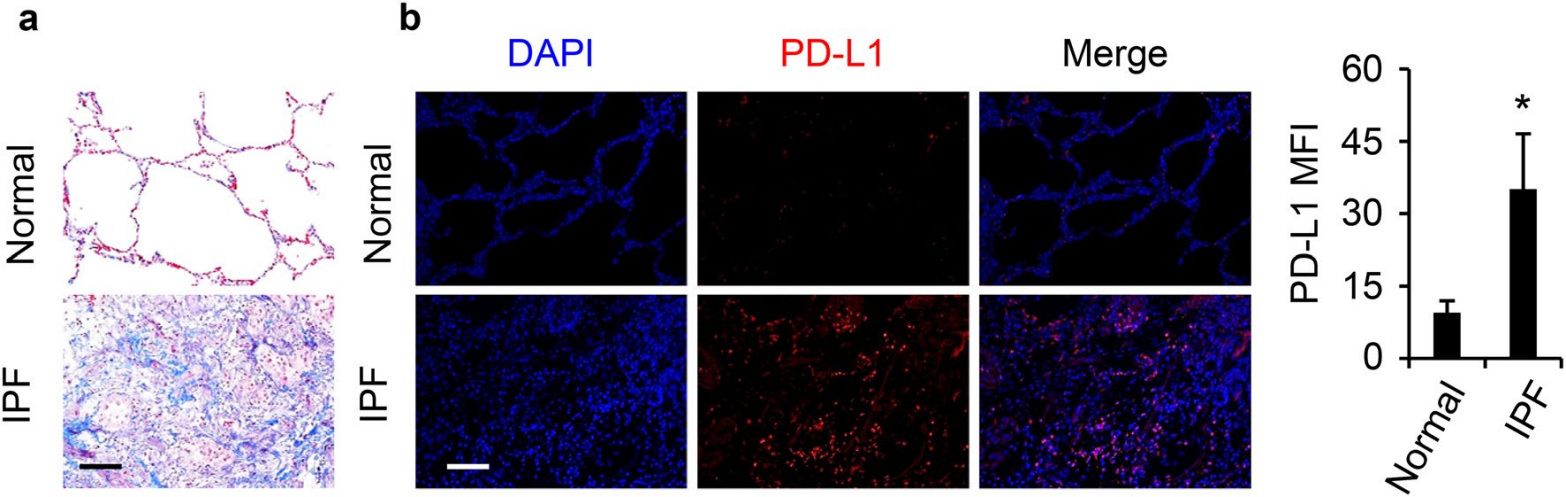
PD-L1 expression was increased in human IPF patients. (**a**) Representative images of Masson’s trichrome staining showed the remodeling of the lung and collagen deposition (blue) associated with IPF (n = 3/group). (**b**) Representative immunofluorescence (IF) staining showed increased PD-L1 (red) levels in the lungs of IPF patients. DAPI stained the nuclei (blue). Bars (black and white) represent 100 µm. The quantification and comparison of PD-L1 mean fluorescence intensity (MFI) was based on measurements from 10 representative fields per slide from each of three normal and IPF patients. *, *P* < 0.05 compared with normal controls.

We first determined if PD-L1 expression is abnormally regulated in the lungs of IPF patients. Compared to control human lung tissues, those from IPF patients showed increased collagen deposition (Fig. 1a). Moreover, PD-L1 level was dramatically increased in IPF patients as shown by immunostaining (Fig. 1b). Quantification and comparison of PD-L1 immunofluorescence intensity showed a significant increase of PD-L1 in IPF tissues versus normal controls (n = 3 per group). The data implicate increased PD-L1 in the pathogenesis of human IPF.

### Elevated PD-L1 levels in experimental models of pulmonary fibrosis

**Figure 2:**
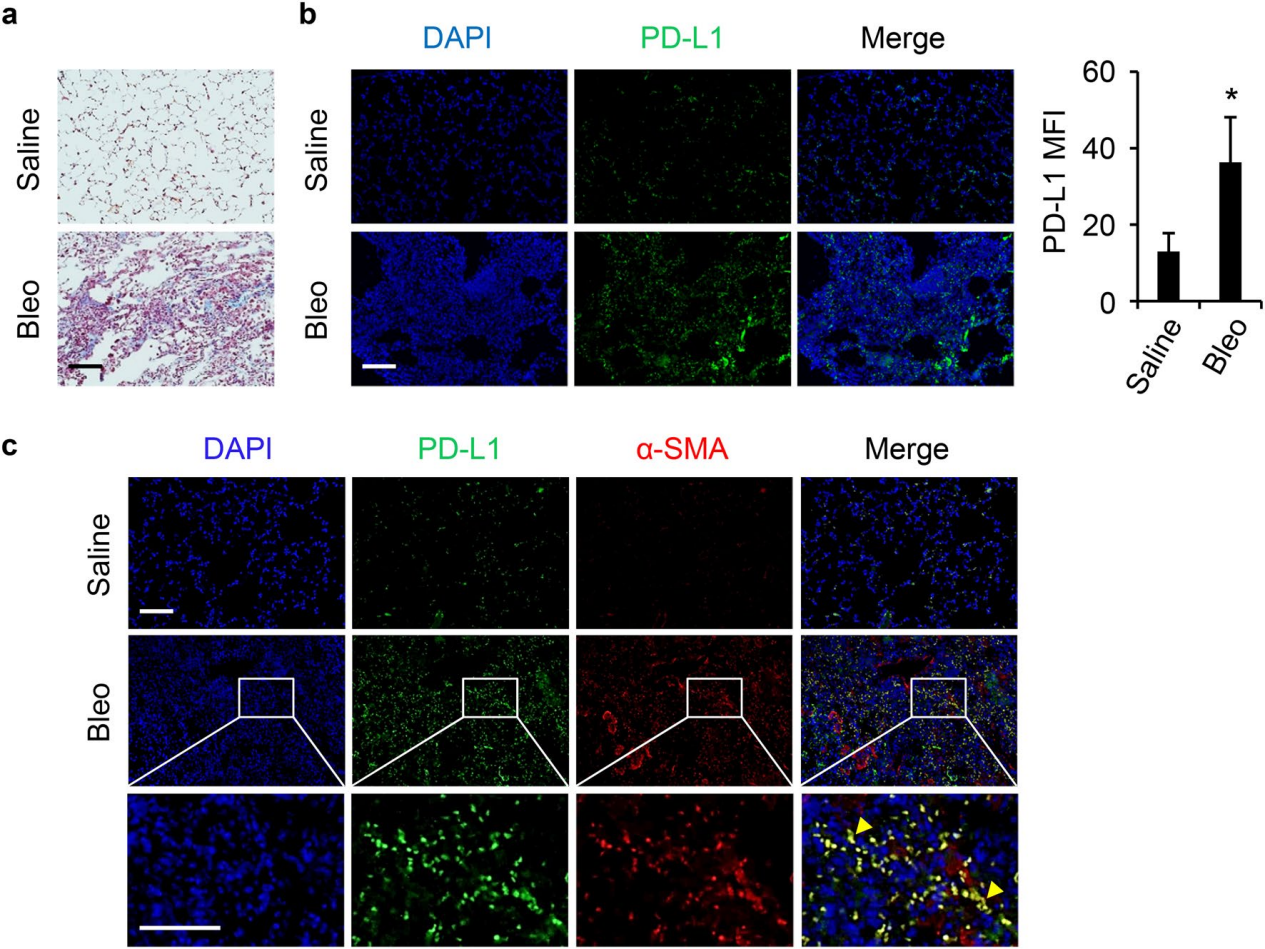
PD-L1 expression was induced in bleomycin induced pulmonary fibrosis in vivo. (**a**) Masson’s trichrome staining showed increased lung collagen deposition in mice given bleomycin (Bleo) for 3 weeks. (**b**) IF staining showed significantly increased lung PD-L1 (green) in mice received intratracheal Bleo. The quantification and comparison of PD-L1 mean fluorescence intensity (MFI) was based on measurements from 10 representative fields per slide/mouse, n = 3 mice per group. MFI, mean fluorescence intensity. *, *P* < 0.05 compared with corresponding control group. (**c**) Representative IF images showing co-staining of PD-L1 (green) and α-SMA (red) in lung tissues of mice received saline or Bleo. Bars (black and white) represent 100 µm. Enlarged views (white squares) are shown below to indicate co-staining of PD-L1 and α-SMA by yellow arrowheads. n = 3 mice per group.

Intratracheal injection of bleomycin is a conventional way to induce pulmonary fibrosis in mice^24^. We found that intratracheal bleomycin treatment induced increased collagen deposition in the mouse lung (Fig. 2a). Compared to the saline control, bleomycin injured mice showed significantly increased PD-L1 levels in the lung (Fig. 2b). In addition, increased expression and colocalization of PD-L1 and α-SMA were observed in the lung tissues of bleomycin injured mice (Fig. 2c). Using archived tissues collected from a previous study^16^, we also found PD-L1 level was significantly increased in the lung tissues from mice intratracheally administered TGF-β adenovirus (Supplementary Fig. 1). These data show that PD-L1 is upregulated in distinct preclinical models of pulmonary fibrosis and suggest that PD-L1 is also linked to the process of FMT.

### TGF-β induces increased levels of PD-L1 in primary normal and IPF HLFs

**Figure 3:**
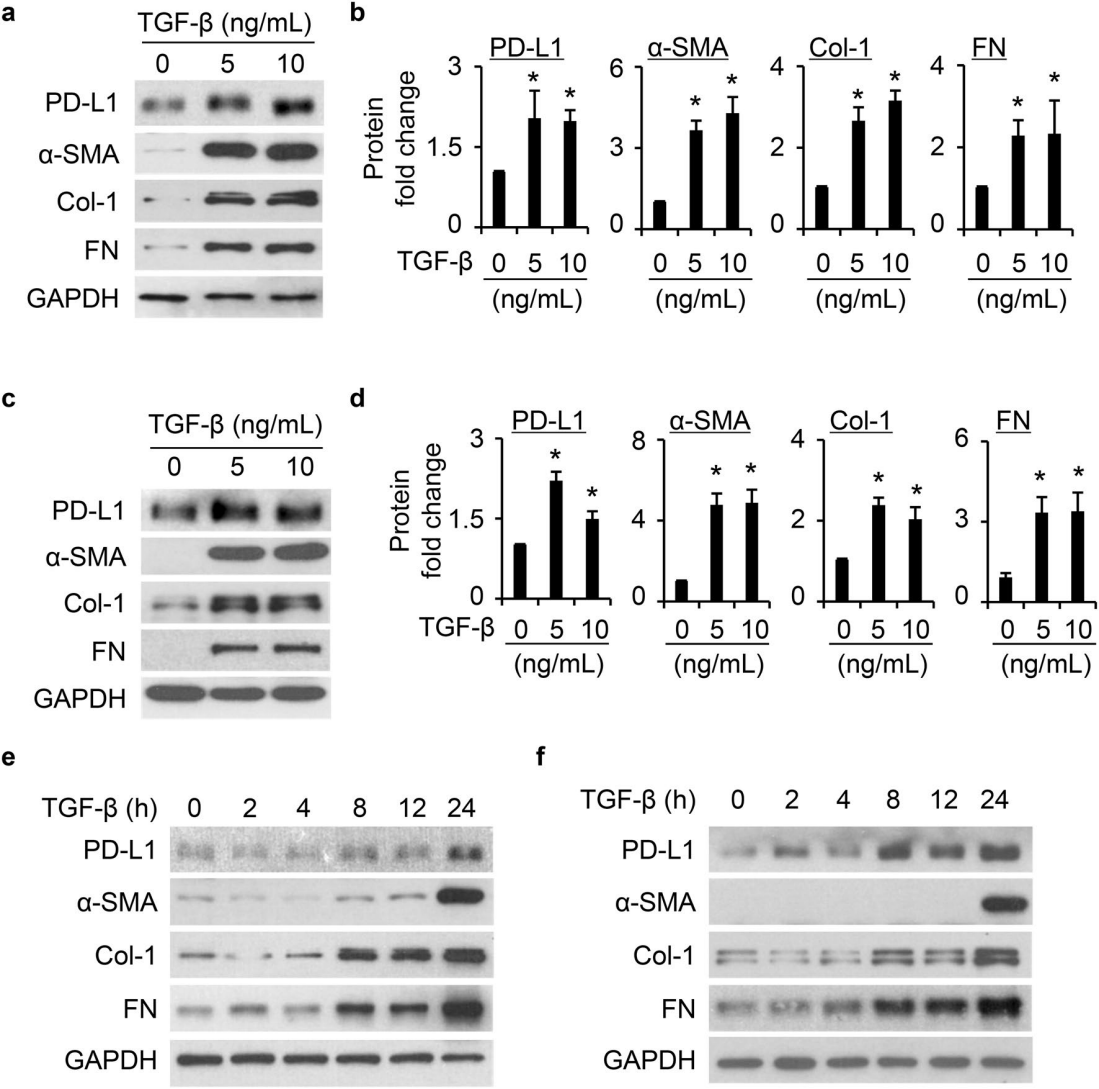
PD-L1 expression was induced by TGF-β in primary normal and IPF HLFs. (**a**) and (**c**), TGF-β significantly induced increase of PD-L1 levels along with α-SMA, collagen type 1 (Col-1), and fibronectin (FN) in both primary normal (**a**) and IPF (**c**) HLFs. (**b**) and (**d**) are quantification of (**a**) and (**c**), respectively, based on three independent experiments. (**e**) and (**f**), TGF-β (5 ng/mL) induced PD-L1 levels in a time dependent manner in primary normal (**e**) and IPF (**f**) HLFs. *, *P* < 0.05 compared with vehicle control.

We next tested whether TGF-β induces PD-L1 in primary HLFs. TGF-β potently induces increased level of PD-L1 at 5 ng/mL and 10 ng/mL in normal (Fig. 3a–b) HLFs. The FMT markers α-SMA, collagen type 1 (Col-1), and fibronectin (FN) were likewise induced by TGF-β. Since the concentration of 5 ng/mL TGF-β was sufficient to induce the expression of FMT markers and PD-L1, it was selected for the subsequent experiments. Temporal evaluation showed that PD-L1 was induced by TGF-β in a time dependent manner, along with the FMT markers α-SMA, Col-1, and FN in normal (Fig. 3e) HLFs. Lung fibroblasts from IPF patients have been thought to be modified epigenetically^25^. We thus also included IPF HLFs into these tests. Similar induction of PD-L1 by TGF-β were observed in IPF HLFs (Fig. 3c–d and f).

### PD-L1 is required for TGF-β induced FMT in primary normal and IPF HLFs

**Figure 4:**
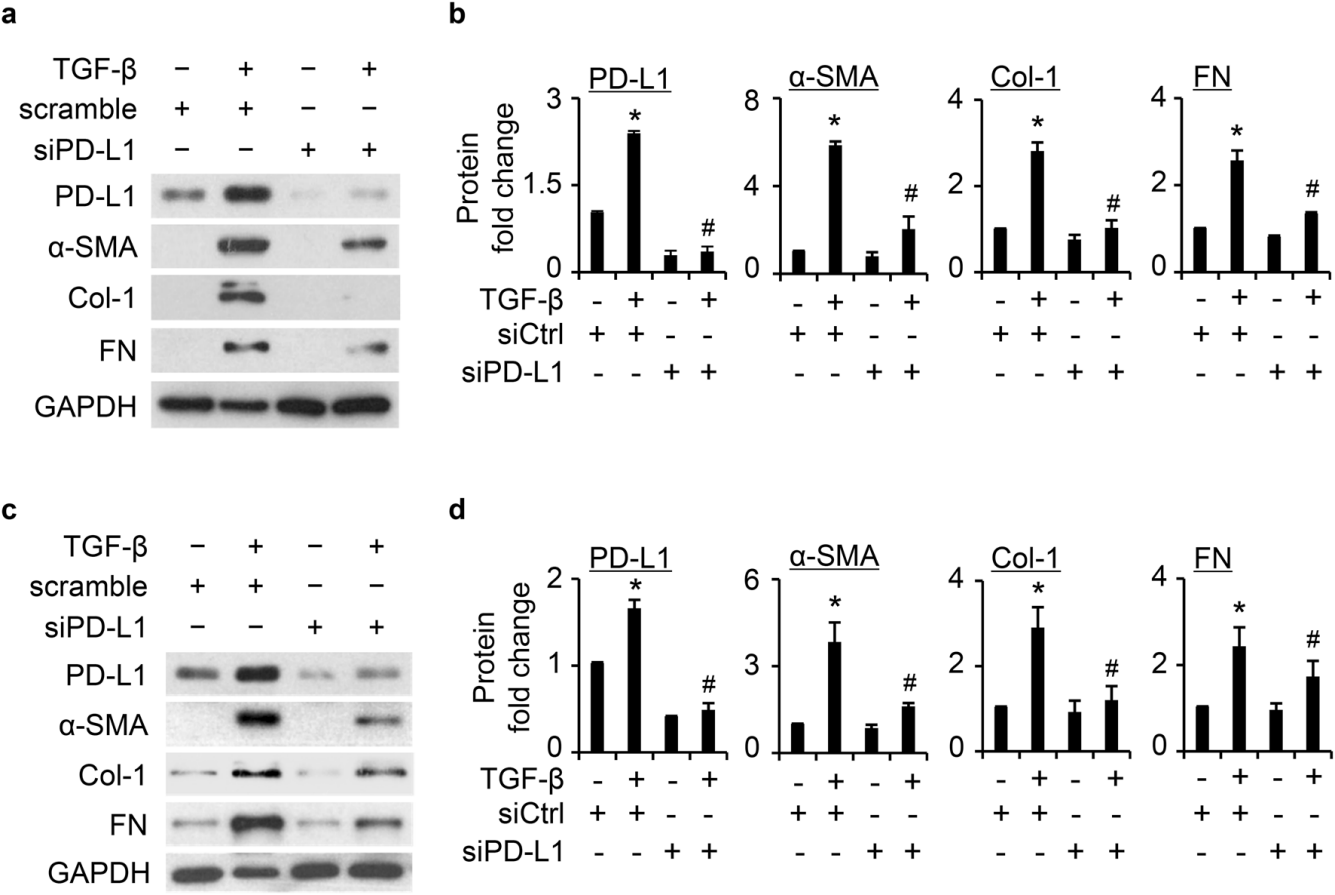
PD-L1 knockdown significantly attenuates TGF-β-induced fibroblast to myofibroblast transition (FMT) in primary normal HLFs. In two lines of primary normal HLFs (**a** and **c**), we first transfected the cells using scramble (siCtrl) or siPD-L1 (40 nM), followed by treatment with TGF-β (5 ng/mL) for additional 24 h to detect PD-L1 and α-SMA, Col-1, and FN levels. (**b**) and (**d**) are quantification of (**a**) and (**c**), respectively, based on three independent experiments. ImageJ software was used to quantify the band intensity. *, *P* < 0.05 vs control group. #, *P* < 0.05 vs. TGF-β-treated scramble control group.

Next, we evaluated the role of PD-L1 in TGF-β-induced FMT. We knocked down PD-L1 in primary HLFs using siRNA, followed by treatment with TGF-β (5 ng/mL) for 24 h. PD-L1 level was significantly reduced by siRNA treatment, even in the presence of TGF-β (Fig. 4a–d). Further, the induction of FMT markers, including α-SMA, Col-1 and FN, were significantly decreased in PD-L1 downregulated cells (Fig. 4a–d). Similar results were observed in two lines of primary normal HLFs (Fig. 4a–d). Similar results were also found in IPF HLFs (Supplementary Fig. 2). Consistently, IF staining showed that knockdown of PD-L1 dramatically suppressed TGF-β-induced α-SMA localization/presence (Supplementary Fig. 3). These data strongly suggest the important role of PD-L1 in TGF-β-induced FMT.

### Transcriptional regulation of PD-L1 by TGF-β is Smad3 and p38 dependent

**Figure 5:**
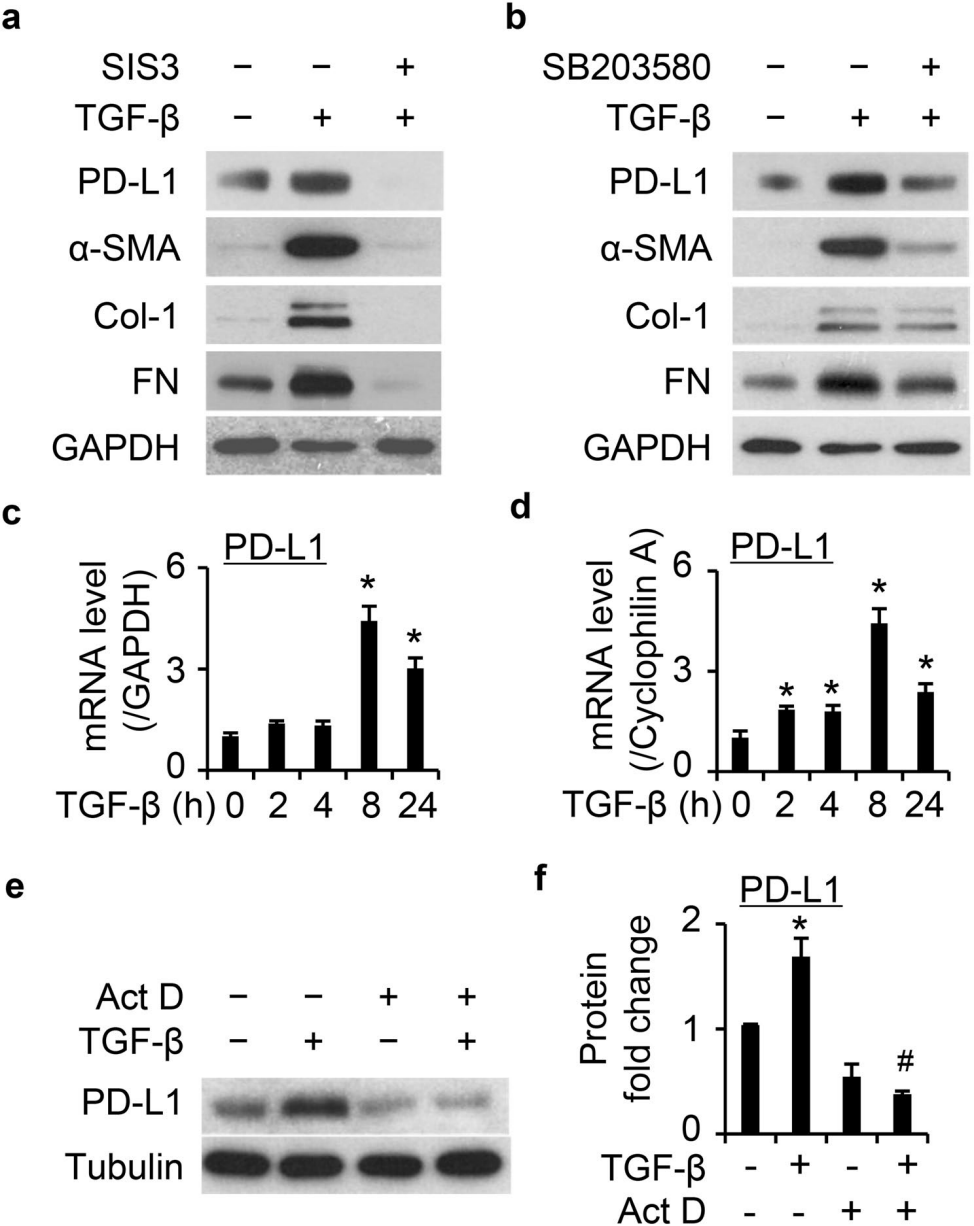
TGF-β upregulates PD-L1 at the transcriptional level and in Smad3 and p38 dependent manner. (**a**) and (**b**) pretreatment with Smad3 inhibitor SIS3 (10 µM) (**a**) or p38 inhibitor SB203580 (10 µM) (**b**) for 30 min abolished/attenuated the induction of PD-L1 and FMT markers in primary normal HLFs. (**c**) qPCR data showed that TGF-β (5 ng/mL) induced PD-L1 mRNA expression in a time dependent manner in primary normal HLFs, which was significantly increased after 8 h treatment. GAPDH was used as a reference gene. (**d**) Similar result was found as in (**c**) using a different reference gene cyclophilin A. (**e**) Pretreatment with 1 µg/mL Actinomycin D (Act D) for 30 min blocked TGF-β (5 ng/mL) induced expression of PD-L1 in the primary normal HLFs. (**f**) The quantification of the data illustrated in (**e**) based on three independent experiments. ImageJ software was used to quantify the band intensity. *, *P* < 0.05 vs. control group. #, *P* < 0.05 vs. TGF-β-treated control group.

Numerous pathways are reported to regulate the level of PD-L1 by TGF-β, including the Smad3 pathway among others^19^. Pretreatment with the Smad3 inhibitor SIS3 (10 µM)^26^ for 30 min blocked TGF-β mediated increase of PD-L1 and the FMT markers (Fig. 5a), which also blocked phosphorylation of Smad3 induced by TGF-β (Supplementary Fig. 4). In Fig. 5b we show that p38 signaling is likewise involved in TGF-β induced upregulation of PD-L1 in primary HLFs. Pretreatment with the p38 inhibitor SB203580 (10 µM)^27^ for 30 min also attenuated PD-L1 induction by TGF-β (Fig. 5b). These data together demonstrate Smad3 activation dependent and independent regulation of PD-L1 by TGF-β.

To further determine the regulation of PD-L1 by TGF-β, we performed qPCR analysis of RNA samples collected from cells treated with vehicle or TGF-β (5 ng/mL) at various time points. In primary HLFs, TGF-β induced PD-L1 mRNA expression in a time dependent manner, peaking at 8 h after treatment and sustaining up to 24 h using *GAPDH* (Fig. 5c) and *cyclophilin A* (Fig. 5d) as internal reference genes. Pre-treatment of the cells with the transcription inhibitor Actinomycin D (Act D) successfully blocked the effect of TGF-β (Fig. 5e–f). Similar results were observed in an additional primary normal HLFs (Supplementary Fig. 5). Together, these data demonstrate the transcriptional regulation of PD-L1 by TGF-β.

### PD-L1 interacts with Smad3 to mediate TGF-β-induced FMT

**Figure 6:**
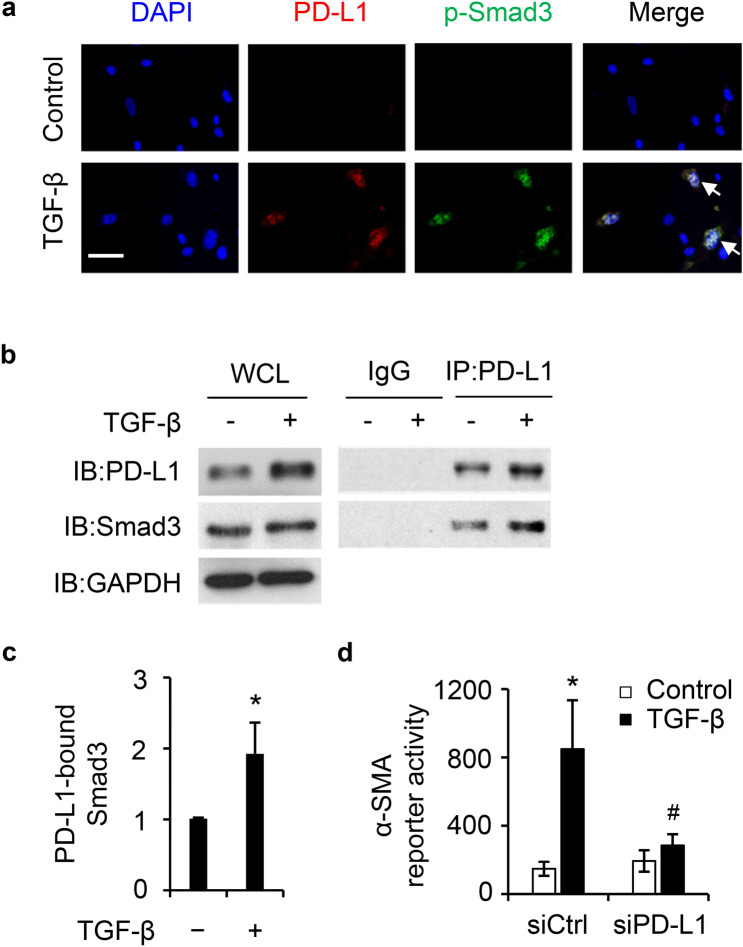
PD-L1 interacts with Smad3 to facilitate transcription of α-SMA upon TGF-β treatment in primary HLFs. IF staining of PD-L1 and phosphor-Smad3 (p-Smad3) showed that TGF-β treatment (5 ng/mL, 16 h) increased expression of PD-L1 and p-Smad3, which largely colocalized in the nuclei (**a**) in primary IPF HLFs. White arrows indicate nuclear co-staining of PD-L1 and p-Smad3. Bar represents 50 µm. (**b**) Co-IP assay showed that Smad3 interacts with PD-L1 and TGF-β treatment (5 ng/mL, 12 h) increased their interaction in primary IPF HLFs using PD-L1 antibody for the pulldown. WCL, whole cell lysate. (**c**) Quantification of PD-L1-bound Smad3 by normalizing to the input Smad3 level in each treatment and set the vehicle-treated group as 1. *, *P* < 0.05 vs the vehicle treated group (-), n = 3 replicates. (**d**) α-SMA reporter vector and scramble (siCtrl) or siPD-L1 were co-transfected into primary normal HLFs for 36 h, followed by TGF-β (5 ng/mL) treatment for an additional 12 h, followed by measurement of luciferase activity, n = 3 replicates. *, *P* < 0.05 vs. scramble control group. #, *P* < 0.05 vs. TGF-β-treated scramble control group.

Smad3 is a potent transcriptional factor for FMT marker gene expression in pulmonary fibrosis and subepithelial fibrosis in asthma^28–32^. In this study we focused on Smad3 and determined the potential effect of PD-L1 on Smad3. PD-L1 has also been reported to translocate to the nuclei to affect different immune response related genes in cancer^21^. In this study we found that TGF-β treatment (16 h) increased PD-L1 level (red, Fig. 6a), which was largely localized in the nuclei. Since Smad3 is a known transcription factor associated with inducing FMT^33^, we sought to test if PD-L1, when induced by TGF-β, interacts with Smad3 to affect FMT in primary HLFs. The co-IP assay was performed using PD-L1 antibody for the pulldown to detect the interaction between PD-L1 and Smad3 in primary IPF HLFs (Fig. 6b–c, Supplementary Fig. 6a and b). TGF-β treatment showed increased interaction between PD-L1 and Smad3. In view of  the interaction between PD-L1 and Smad3, we next tested whether knockdown of PD-L1 affects the binding of Smad3 to the α-SMA promoter using a promoter/reporter assay in primary HLFs. The results show that knockdown of PD-L1 in primary HLFs significantly inhibited TGF-β induced α-SMA promoter activity (Fig. 6d). Together, these data indicate that PD-L1 interacts with Smad3 and may act as a co-transcription factor to induce α-SMA transcription. Additionally, we tested whether manipulation of PD-L1 affects Smad3 activity. We found that PD-L1 knockdown attenuated TGF-β induced phosphorylation of Smad3 in primary normal HLFs (Supplementary Fig. 7).

### The involvement of GSK3β/β-catenin signaling in PD-L1 mediated FMT

**Figure 7:**
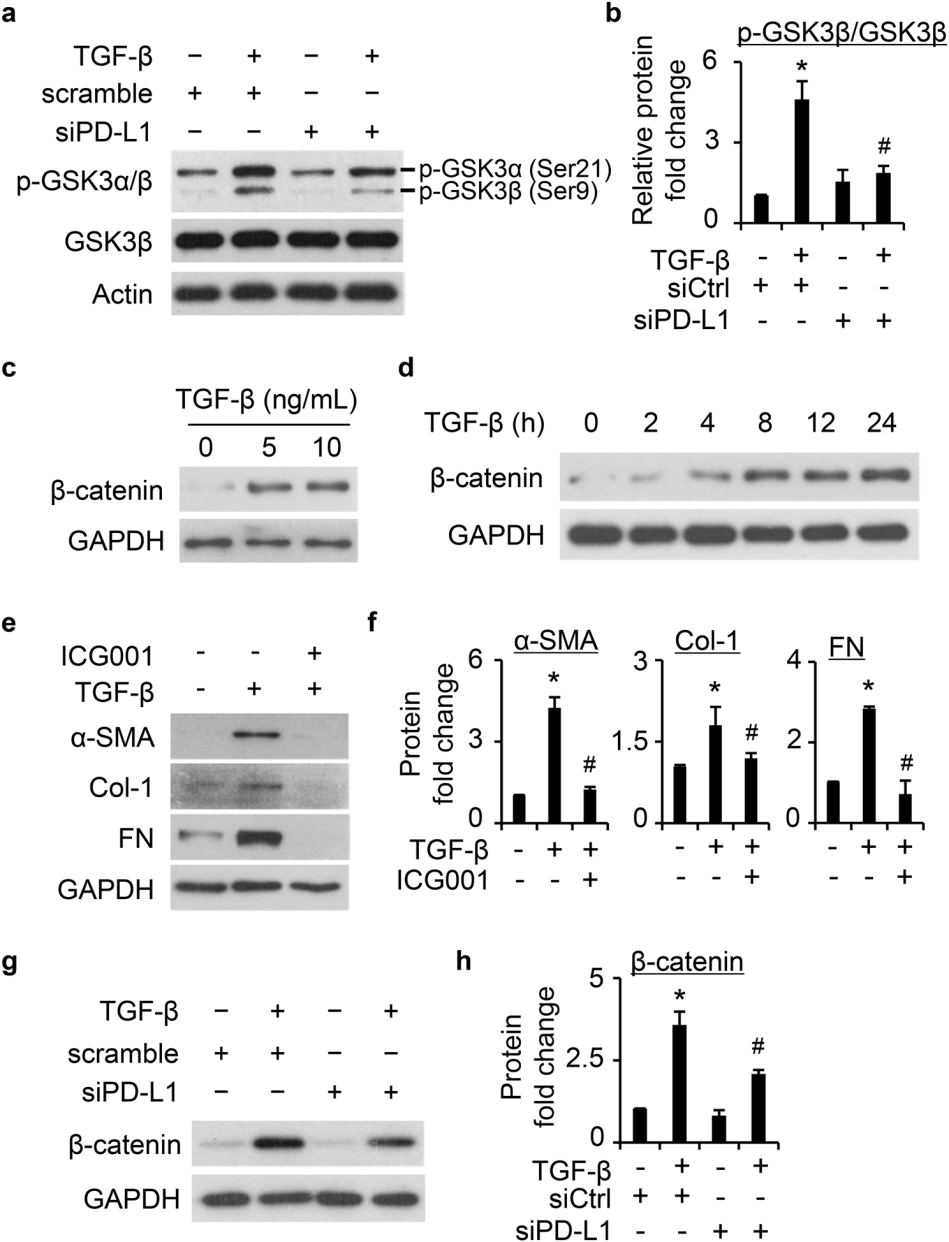
PD-L1 contributes to TGF-β-induced FMT involving the GSK3β/β-catenin pathway. (**a**), TGF-β (5 ng/mL, 24 h) induced phosphorylation of GSK3β (Ser9) and knockdown of PD-L1 using siRNA (siPD-L1) significantly attenuated GSK3β phosphorylation. (**b**), Quantification of a based on three independent experiments. (**c**), TGF-β induced β-catenin expression at 5 and 10 ng/mL in primary normal HLFs. (**d**), TGF-β (5 ng/mL) induced β-catenin expression in a time dependent manner in normal primary HLFs. (**e**), Pretreatment with the β-catenin inhibitor ICG001 (10 µM) for 30 min significantly blocked TGF-β (5 ng/mL) induced FMT marker expression. (**f**), Quantification of the data illustrated in (**e**) based on three independent experiments. (**g**), Knockdown of PD-L1 significantly attenuated TGF-β (5 ng/mL, 24 h) induced β-catenin expression. (**h**), Quantification of g based on three independent experiments. *, *P* < 0.05 vs. control group. #, *P* < 0.05 vs. TGF-β-treated scramble control group.

A recent study showed that PD-L1 activates β-catenin signaling to promote lung cancer cell progression^22^. The GSK3β/β-catenin signaling is also known to regulate FMT as a non-canonical pathway of TGF-β^34,35^. We next evaluated whether this pathway is involved in PD-L1 mediated FMT. First, we show that TGF-β increased the inhibitory phosphorylation of GSK3β at Serine 9 (Ser9) in normal HLFs (Supplementary Fig. 8). Further, knockdown of PD-L1 significantly attenuated TGF-β induced GSK3β Ser9 phosphorylation (Fig. 7a–b). Next, we determined the induction of β-catenin, a known target of GSK3β, by TGF-β. TGF-β treatment enhanced β-catenin protein levels at 5 ng/mL and no further increase was found at 10 ng/mL (Fig. 7c), similarly as the induction of FMT markers (Fig. 3c). A time dependent induction of β-catenin was also observed (Fig. 7d). Inhibition of β-catenin signaling with the specific inhibitor ICG001 (10 µM)^36^ significantly attenuated TGF-β-induced FMT marker expression (Fig. 7e–f), indicating the involvement of β-catenin signaling in TGF-β induced FMT. PD-L1 downregulation by siRNA also significantly attenuated the induction of β-catenin by TGF-β in primary HLFs (Fig. 7g–h). Together, these data suggest that PD-L1 mediates TGF-β induced FMT at least partly through modulation of the GSK3β/β-catenin signaling axis.

### A proposed model by which PD-L1 mediates FMT induced by TGF-β

**Figure 8:**
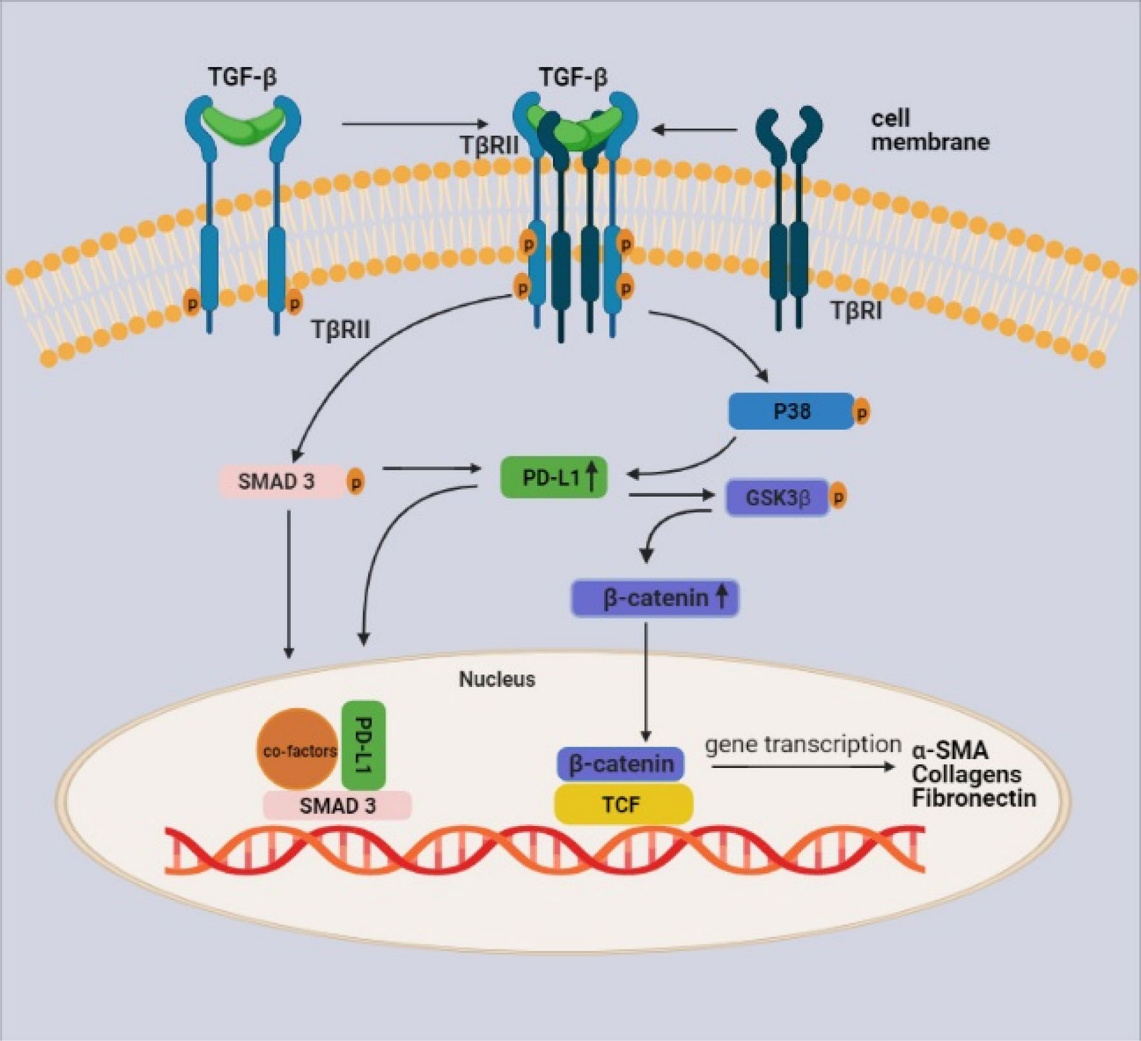
Schematic view of the proposed mechanisms by which PD-L1 mediates FMT induced by TGF-β in HLFs. The binding of active TGF-β with cell surface receptors (TGF receptor I and II, TβRI and TβRII) activates the receptor kinase which further recruits and phosphorylates Smad3. Activated Smad3 then translocate into the nucleus where it binds with other co-factors to activate transcription of FMT related genes. PD-L1 can be increased by Smad3 and p38 pathways downstream of TGF-β. Increased PD-L1 further binds to Smad3 and enhance its transcription activation of FMT genes including α-SMA. In addition, PD-L1 also facilitates GSK3β phosphorylation at Ser9 and thus inhibiting GSK3β dependent degradation of β-catenin. Increased β-catenin may also contribute to TGF-β induced FMT gene expression through binding with the transcription factor, T cell factor (TCF).

Based on the data as mentioned above, we proposed a model that might account for the role of PD-L1 in mediating TGF-β induced FMT (Fig. 8). Binding of active TGF-β to its type II receptor results in phosphorylation at the intracellular kinase domain, which further recruits type I receptors to fully activate downstream signaling. Both the canonical and non-canonical Smad pathways are activated as a result. Smad3 and p38 activation result in upregulation of PD-L1 in HLFs. The increased PD-L1 binds to Smad3 and further facilitates its transcriptional activity in the nucleus. On the other hand, PD-L1 also enhances TGF-β-induced phosphorylation of GSK3β at Serine 9 thereby inhibiting the degradation of β-catenin mediated by GSK3β, resulting in upregulation of β-catenin. Accumulated β-catenin then translocate into the nucleus where it binds and promotes the activity of the transcriptional factor T cell factor (TCF), contributing to FMT target gene expression. α-SMA is shown as one of the target genes that are unregulated by Smad3 and the β-catenin pathway.

## Discussion

IPF remains a devasting disease with poor prognosis and the mechanisms that govern its progression remain incompletely understood. While two recent reports delineate the important role of PD-L1 in lung fibroblasts in the pathogenesis of pulmonary fibrosis^19,20^, the underlying mechanisms have remained obscure. In this study, we found that PD-L1 is not only induced by TGF-β in human primary normal and IPF HLFs but it is also required for TGF-β-induced FMT, a critical event in the development of pulmonary fibrosis. We found that both the canonical Smad pathway and non-canonical pathway (e.g., p38 signaling) are activated and result in upregulation of PD-L1 in HLFs. We also elucidated previously undefined mechanisms by which PD-L1 contributes to FMT, i.e., through interaction with Smad3 to promote its transcription activity, and through activation of the β-catenin pathway. These findings provide novel insight into the role and mechanisms of PD-L1 in the pathogenesis of pulmonary fibrosis.

PD-L1 as an immune checkpoint is critical in immune tolerance and preventing autoimmune response. Tumor cells utilize increased PD-L1 expression to escape from immune surveillance^37^ and to gain resistance for therapeutic agents such as tyrosine kinase inhibitors^38^. The similarities between cancer and IPF are increasingly recognized, and certain treatment options for cancer have been considered for IPF including the anti-PD-1/PD-L1 therapy^7,8^. However, understanding of PD-L1’s role as a pathological factor or therapeutic target for IPF is still in its infancy. In this study, we observed significantly increased PD-L1 expression in the lung tissues of IPF patients compared to normal subjects (Fig. 1b), suggesting a role for PD-L1 in the pathogenesis of IPF. We also found that PD-L1 expression was upregulated in the bleomycin induced preclinical PF model (Fig. 2b) as well as in the TGF-β preclinical model (Fig. 2d), further supporting that PD-L1 is an integral part of the development of PF. We also noted that those cells with elevated PD-L1 expression were also positive for α-SMA in the lung tissues of mice challenged with bleomycin but not saline (Fig. 2c), suggesting that PD-L1 may affect the FMT during PF progression in vivo.

Increased infiltration of immune cells has been repeatedly observed in the lungs of IPF patients^39,40^ raising the hypothesis that immune deregulation is a driver for the pathophysiology of IPF^41^. Recent evidence strengthens a crucial role of the PD-1/PD-L1 axis in promoting PF. Using PD-1 null mice or antibody-based neutralization strategies to block PD-L1 significantly attenuated bleomycin induced PF. Further, PD-1^+^CD4^+^T cells were increased in IPF patients with increased expression of TGF-β and IL17A^12^, strong inducers of profibrotic changes. On the other hand, PD-L1 expression in lung fibroblasts have been reported in several studies^13,19,20^. Higher PD-L1 expression in a subset of HLFs is associated with an invasive phenotype and drives the development of PF in a humanized mouse model^20^. The findings here directly support fibroblast intrinsic PD-L1 as an important pathological factor in the development of PF. While higher expression of PD-L1 in primary HLFs is linked with higher invasive capacity^20^, the induction and role of PD-L1 by profibrotic cytokines such as TGF-β in primary normal and IPF HLFs remains undefined. A recent paper shows that TGF-β induced PD-L1 and the latter is involved in the production of extracellular matrix induced by TGF-β^19^. Instead of using established human lung fibroblast cell lines, we utilized primary normal and IPF HLFs to determine whether PD-L1 is required for FMT thus contributes to PF development in the current study. We found that TGF-β induced PD-L1 expression at both protein and mRNA levels in a time dependent manner in different primary normal and IPF HLFs, along with the induction of FMT markers α-SMA, Col-1, and FN (Fig. 3).

We further showed that knockdown of PD-L1 significantly attenuated TGF-β-induced expression of FMT markers, α-SMA, Col-1, and FN in two lines of primary normal (Fig. 4) and IPF (Supplementary Fig. 2) HLFs. These data suggest that PD-L1 is required in the transition from fibroblast to myofibroblast. One recent paper showed similar results in human and murine lung fibroblast lines^19^ and that both Smad dependent and independent pathway inhibitors attenuated the induction of PD-L1 by TGF-β. We also found the induction of PD-L1 by TGF-β is mediated by both Smad3 and p38 pathway in primary normal LFs (Fig. 5), suggesting both Smad3 activation dependent and independent p38 regulation of PD-L1 by TGF-β in LFs. Interestingly, we also observed that PD-L1 knockdown appears to inhibit TGF-β induced FMT more prominent at 24 h than 48 h in primary normal HLFs (Supplementary Fig. 9), which might be related to the transit effect of siRNA treatment. Further research using PD-L1 knockout cell lines is needed to explore whether PD-L1 deficiency blocks FMT over a longer time.

It has been reported that PD-L1 expression in lung fibroblasts drives cell migration and invasion^20^ as well as production of extracellular matrix proteins^19^. Beyond that, little is known about how PD-L1 expression in lung fibroblasts contributes to the development of PF. Our novel study shows that PD-L1 acts through both Smad3 dependent and independent pathways to promote FMT induced by TGF-β. Further, Smad3 is a crucial transcription factor responsible for TGF-β-induced PF, as Smad3 KO mice were protected from bleomycin induced PF^42^. PD-L1 has also been reported to express in the nuclei of certain cancer types^21,43^. We found that TGF-β treatment increased levels of PD-L1 in the nuclear compartment (Fig. 6a). We further speculated that PD-L1 may act as a transcriptional cofactor of Smad3 to enhance FMT gene expression, such as α-SMA. Indeed, we found that Smad3 co-immunoprecipitated with PD-L1 and TGF-β treatment increased their interaction (Fig. 6b–c, Supplementary Fig. 6). Furthermore, knockdown of PD-L1 significantly decreased the activation of α-SMA reporter activity induced by TGF-β (Fig. 6d). Taken together, these data support that PD-L1 might act as a co-transactivator of TGF-β to induce FMT gene expression. Interestingly, we also found that PD-L1 knockdown attenuated the activation of Smad3 (Supplementary Fig. 7). It remains to be defined whether the binding of PD-L1 to Smad3 affects its activation or stability, for which further research is needed.

Nuclear translocation of PD-L1 was recently reported to be mediated by the histone acetyltransferase p300 in cancer cells^21^. p300 has been found to mediate TGF-β signaling by binding to Smad3 and transactivates gene expression^44,45^. A recent study further suggests that p300/CBP may act as a coactivator to induce PD-L1 transcription in prostate cancer^46^. Therefore, it is likely that in lung fibroblasts TGF-β also activates p300 to increase the acetylation of PD-L1 resulting in its nuclear translocation, which is a topic for dedicated future study.

It has been reported that PD-L1 upregulation mediates cancer progression through activating the β-catenin pathway^22^. Whether such regulation also occurs in lung fibroblast remains undefined. GSK3β/β-catenin signaling deregulation has been shown to contribute to the development of pulmonary fibrosis^23^. In the present study, we found that PD-L1 downregulation suppresses β-catenin induction by TGF-β. Further, inhibition of β-catenin signaling with the specific inhibitor ICG001 suppressed TGF-β induced FMT marker expression, implying the involvement of β-catenin signaling in TGF-β-induced FMT in primary HLFs. PD-L1 was also found to participate in TGF-β induced phosphorylation and inhibition of GSK3β at Serine 9, therefore inhibiting β-catenin degradation. Consistently, TGF-β induced β-catenin upregulation at protein level but not at the mRNA level (data not shown). Further, downregulation of PD-L1 also significantly inhibited TGF-β induced upregulation of β-catenin. These several lines of evidence suggest that PD-L1 may also mediate TGF-β induced FMT through the β-catenin signaling.

TGF-β induced increased levels of β-catenin in primary HLFs and the inhibition of β-catenin signaling significantly decreased TGF-β induced expression of α-SMA, Col-1, and FN (Fig. 7b–c), suggesting that β-catenin is activated as a non-canonical pathway to mediate FMT, consistent with previous findings^47^. TGF-β modulates β-catenin pathway through different mechanisms. A previous study showed that TGF-β targets β-catenin through inhibition of GSK3β via ERK activation in HLFs, which contributes to FMT induced by TGF-β^48^. In lung cancer cells, PD-L1 was also found to upregulate β-catenin through PI3K/AKT/GSK3β pathway^22^. Both studies suggest a posttranslational regulation of β-catenin by TGF-β. In accord with this, we did not find the elevation of β-catenin mRNA levels upon TGF-β treatment at different time points in LFs (data no shown). TGF-β was also reported to activate the Wnt/β-catenin signaling through downregulating the expression of the Wnt antagonist Dickkopf-1^47^. The detailed mechanism is beyond the current study. Based on these above findings, we propose a model for how PD-L1 mediates TGF-β induced FMT (Fig. 8). The binding of active TGF-β to its receptors phosphorylates and activates Smad3 and p38 pathways, both increase PD-L1 levels in HLFs. Upregulated PD-L1 in turn interacts with Smad3 and promotes Smad3-mediated transcriptional upregulation of myofibroblast maker genes including *α-SMA*. On the other hand, PD-L1 facilitates phosphorylation of GSK3β (Ser9) induced by TGF-β, leading to accumulation of β-catenin, which then translocate into the nucleus where it acts as a co-transcriptional factor to further upregulate myofibroblast marker gene expression. Recent publications show that Smad and p38 pathways play a key role in the subepithelial fibrosis associated with asthma, which is closely related to promoting FMT of human bronchial fibroblasts^49,50^. Interestingly, our study here indicates that both pathways are responsible for upregulating the levels of PD-L1 and that PD-L1 is an important mediator of FMT. Together, these findings suggest that PD-L1 is likely an important mediator of FMT and thus a potential target in the development of subepithelial fibrosis in asthma patients. Future research is warranted to explore the targeting of PD-L1 as a novel approach for hampering myofibroblast formation and subepithelial fibrosis in the context of asthma.

In summary, we found that PD-L1 is implicated in the pathogenesis of pulmonary fibrosis in both IPF lung tissues and experimental models of pulmonary fibrosis. In primary HLFs, PD-L1 mediates TGF-β-induced FMT through both Smad3 dependent and independent pathways. Targeting PD-L1 may eventually offer therapeutic benefit for the treatment of IPF.

## Materials and methods

### Reagents and antibodies

Recombinant human transforming growth factor-β1 (TGF-β, 7754-BH-025/CF) was purchased from R&D Systems, Inc. (Minneapolis, MN). Bleomycin (15 Unit/vial, Teva) was purchased from the Pharmacy of the University of Texas Health Science Center at Tyler. Small molecular inhibitors including SIS3 (HY-13013), SB230580 (HY-10256), ICG001 (HY-14428) and actinomycin D (Act D, HY-17559) were purchased from MedChemExpress (Monmouth Junction, NJ). The primary antibodies used in the study includes PD-L1 (clone E1L3N and 405.9A11, Cell Signaling Technology), Smad3 (clone 38-Q, Santa Cruz Biotechnology), phosphor-Smad3 (9520S, Cell Signaling Technology), α-SMA (clone 1A4, Sigma), Collagen type 1 (1310-08, SouthernBiotech)^16^, Fibronectin (ab2413, Abcam), phosphor-GSK3α/β (Ser21/Ser9, 9331S, Cell Signaling Technology), GSK3β (12456S, Cell Signaling Technology), β-catenin (Cat#sc-7963, Santa Cruz), Actin (A2066, Sigma), and GAPDH (1E6D9, Proteintech).

### Human lung tissues from normal and IPF patient

Lung tissue sections from normal and IPF patients used in this study were provided by the University of Michigan (Ann Arbor, Michigan) under a Material Transfer Agreement. Three control and three IPF lung tissue sections were used for comparison of PD-L1 expression. The IPF patients’ information: patient #1 (51 years old, female, non-smoker), patient #2 (67 years old, male, non-smoker), and patient #3 (63 years old, male, non-smoker). The control subjects’ information: donor #1 (60 years old, male, non-smoker), donor #2 (7 years old, male, non-smoker), and donor #3 (59 years old, male, smoker with asthma but not IPF). All methods were carried out in accordance with relevant guidelines and regulations and research protocols were approved by the Michigan Medicine Institutional Review Board. Informed consent was obtained from all subjects.

### Bleomycin/TGF-β-induced PF in mice

C57BL/6 mice (12–16 weeks old, male) were administered bleomycin (1 U/kg, Teva) or 0.9% saline once through intratracheal instillation as previously reported^16^. Bleomycin or saline was given at a volume of 40 µl/mouse. Following bleomycin treatment, mice were monitored and maintained for three weeks. The archived mouse lung tissues from TGF-β adenovirus induced pulmonary fibrosis were reported previously^16^, which were used in this study to examine the induction of PD-L1. The C57BL/6 mice (10–12 weeks old) were purchased from Jackson Laboratory (Bar Harbor ME). A diagrammatic view of the models was shown in Supplementary Fig. 10. All animal experiments were approved by the Institutional Animal Care and Use Committee at the University of Texas Health Science Center at Tyler and performed following the relevant guidelines. All methods are reported in accordance with ARRIVE guidelines for the reporting of animal experiments.

### Cell culture and treatment

The primary normal and IPF human lung fibroblasts (HLFs) were provided by the University of Michigan (Ann Arbor, Michigan) under a Material Transfer Agreement. These cells were maintained in DMEM medium supplemented with 10% fetal bovine serum (FBS) and antibiotics, as previously reported^16^. These cells were used within a passage number of less than ten. The cells were starved in serum free medium overnight before treatment with TGF-β.

### Western blotting

The method for Western blotting is as previously described with minor modifications^51^. Briefly, whole cell lysates were collected from cells after treatments in RIPA lysis buffer containing proteinase inhibitor cocktail and phosphatase inhibitor. A bicinchoninic acid (BCA) protein assay kit was used to determine total protein concentrations according to manufacturer’s guide using the Infinite M Plex plate reader from Tecan (Männedorf, Switzerland). The denatured proteins were used for SDS-PAGE gel electrophoresis, followed by transfer to PVDF membrane. The membrane was blocked with 5% skimmed milk and was cut into several parts based on molecular weight to incubate with different primary antibodies at 4 °C overnight with gentle shaking. Appropriately diluted HRP secondary antibodies were used to detect target proteins including anti-rabbit IgG-HRP (31460) and anti-mouse IgG-HRP (31430) that were purchased from Fisher Scientific (Waltham, MA).

### Co-immunoprecipitation (coIP) assay

The method of coIP assay was reported previously^52^. Briefly, serum-starved HLFs were treated with vehicle or TGF-β (5 ng/mL) for 12 h, followed by collection of whole cell lysates in RIPA buffer supplied with protease inhibitor cocktail and phosphatase inhibitor. The protein concentration was measured and adjusted to equal concentration with RIPA lysis buffer with protease and phosphatase inhibitors. A portion of the lysates were used as whole cell lysates (WCL). The lysates from control and TGF-β treated group were then incubated with either IgG (Cat#3703, ProSci) or PD-L1 antibody (E1L3N, Sigma) for 2 h at 4 °C with rotation. After incubation with these antibodies, Protein A/G PLUS-Agarose (sc-2003, Santa Cruz Biotechnology) was added to each lysate group after pre-wash five times with RIPA buffer without proteinase or phosphatase inhibitor. Then, the mixtures were incubated at 4 °C for 1.5 h with gentle rotation, followed by five times of washing with RIPA lysis buffer without protease or phosphatase inhibitor and once with 50 mM HEPES buffer. The pulldown proteins were used for denatured SDS-PAGE electrophoresis similar as described in Western blotting.

### Luciferase reporter assay

The luciferase assay was conducted similarly to previously described^53,54^. Briefly, primary HLFs were transfected with α-SMA reporter plasmids with scramble or PD-L1 siRNA from Sigma (St. Louis, MO) in a 24-well plate, followed by collection of lysates 12 h after TGF-β treatment. The luciferase activity measurement was done according to the instructions of the luciferase assay kit from Promega (Madison, MI). The measurement of luciferase activity was performed using a Sirius 2 luminometer (Berthold Technologies USA, Oak Ridge, TN). The readings were adjusted by the corresponding protein concentrations and data were shown as relative numbers to the control group.

### RNA extraction and quantitative real-time PCR (qPCR) analysis

Total RNA was extracted from cells after treatment using TRIzol reagent according to the manufacturer’s guide. After quantification of RNA using the Infinite M Plex plate reader from Tecan, 1 µg total RNA was used for reverse transcription to produce cDNA using a reverse transcription kit from BioRad (Hercules, CA). Diluted cDNA templates were then used for qPCR analysis of target gene expression using the SYBR reagent from BioRad. GAPDH was used as the internal control and each sample was run in triplicate. The primers of genes tested were described as reported previously^55,56^ and shown in Supplementary Table 1.

### Lung histology, Masson’s Trichrome staining, and immunofluorescence (IF) staining

Formalin-fixed and paraffin-embedded (FFPE) lung sections from human subjects (normal and IPF patients) and mouse models of pulmonary fibrosis were used for histological and immunological analyses. These sections were undergone serial steps of hydration, followed by H&E staining and collagen staining (Masson’s Trichrome method) according to the manufacturers’ guide. The IF staining was performed similarly as previously reported^53^. Briefly, the hydrated slides were quenched in 3% H_2_O_2_ for 15 min, followed by antigen retrieval using EDTA buffer. The sections were cooled before blocking with 10% goat serum. Then, the sections were incubated with primary PD-L1 antibody (E1L3N, Sigma) overnight at 4 °C. Secondary antibodies used were goat anti-Rabbit/Mouse Alexa Fluor Plus 488 (A32731, Invitrogen) and goat anti-Rabbit/Mouse Texas Red (Cat#2015320, Invitrogen). The images were taken using a Nikon NiU microscope.

### Gene knockdown with small interfering RNA (siRNA)

For knockdown of the expression of PD-L1 in primary HLFs, scramble (SIC001) or siRNA targeting PD-L1 (siPD-L1, SASI_Hs01_00010201) purchased from Sigma (St. Louis, MO) were used. siRNA targeting human β-catenin (sc-29209) was purchased from Santa Cruz Biotechnology (Dallas, TX). Cells were seed in 6-cm dish at a confluence of approximate 50% before transfection with scramble or siPD-L1 using the jetPRIME from Polyplus transfection (New York, NY) as the transfection reagent according to the manufacturer’s guide. Cells were then starved in serum-free DMEM medium overnight before the treatment with vehicle or TGF-β as indicated in the results.

### Statistical analysis

The comparisons were conducted using either Student’s t-test or one-way analysis of variance followed by Fisher’s least significant difference test. Data are shown as mean ± SD. A *P *value less than 0.05 was considered statistically significant.

## Supplementary Information

Supplementary Information.

## Abbreviations

Act D: Actinomycin D
BCA: Bicinchoninic acid
FFPE: Formalin-fixed and paraffin-embedded
FMT: Fibroblast to myofibroblast transition
HEPES: 4-(2-Hydroxyethyl)-1-piperazineethanesulfonic acid
IF: Immunofluorescence
IPF: Idiopathic pulmonary fibrosis
LF: Lung fibroblast
PD-1: Programmed death protein 1
PD-L1: Programmed death ligand-1
PF: Pulmonary fibrosis
RIPA: Radioimmunoprecipitation assay
TGF-β: Transforming growth factor-β
WCL: Whole cell lysate
