# Supplementary material for: PD-L1 mediates lung fibroblast to myofibroblast transition through Smad3 and β-catenin signaling pathways
Source: Sci Rep. 2022 Feb 23;12:3053. doi: 10.1038/s41598-022-07044-3 (PMC8866514; doi:10.1038/s41598-022-07044-3)

PD-L1 mediates lung fibroblast to myofibroblast transition through Smad3 and β-catenin signaling pathways

Xia Guo[1#], Christudas Sunil[1#], Oluwaseun Adeyanju[1], Andrew Parker[1], Steven Huang[2], Mitsuo Ikebe[1], Torry A. Tucker[1,3], Steven Idell[1,3], and Guoqing Qian[1*]

[1]Department of Cellular and Molecular Biology, The University of Texas Health Science Center at Tyler, Tyler, TX, USA; [2]Department of Internal Medicine, Division of Pulmonary and Critical Care Medicine at the University of Michigan; [3]The Texas Lung Injury Institute, Tyler, TX, USA

[#]: These authors contributed equally to this manuscript.

***Correspondence**: Dr. Guoqing Qian, PhD. Department of Cellular and Molecular Biology, University of Texas Health Science Center at Tyler, 11937 US Highway 271, Tyler, TX, 75708. E-mail: guoqing.qian@uthct.edu. Phone: (903) 877-8358.

Supplemental Materials:

Supplemental Table 1. qPCR primers used in the study.

**Genes**		**Primer sequences**
PD-L1	Forward	5’- GGCATTTGCTGAACGCAT-3’
	Reverse	5’- CAATTAGTGCAGCCAGGT-3’
GAPDH	Forward	5'-TGCACCACCAACTGCTTA-3'
	Reverse	5'-GGATGCAGGGATGATGTTC-3'
Cyclophilin A	Forward	5'-GGCAAATGCTGGACCCAACACA-3'
	Reverse	5'-TGCTGGTCTTGCCATTCCTGGA-3'

Supplementary Figure 1.

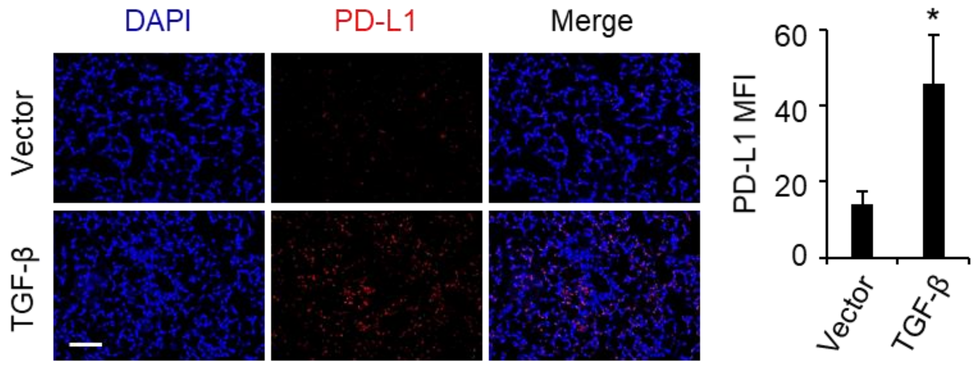

PD-L1 expression was induced in the TGF-β model of pulmonary fibrosis *in vivo*. Intratracheally administered TGF-β adenovirus significantly induced PD-L1 (red) expression in mice lungs compared to adenoviral vector control. Bar (White) represents 100 μm. The quantification is similar as described in Figure 1. MFI, mean fluorescence intensity. *, $P < 0.05$ compared with corresponding control group.

Supplementary Figure 2.

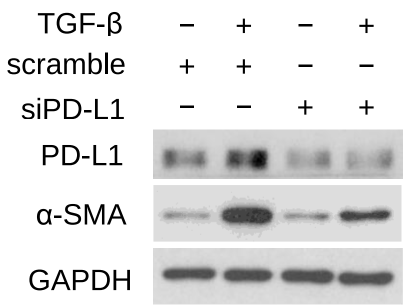

PD-L1 knockdown significantly attenuates TGF-β-induced fibroblast to myofibroblast transition (FMT) in primary IFP HLFs. The IPF HLFs were transfected with scramble or siPD-L1 (40 nM), followed by treatment with TGF-β (5 ng/mL) for additional 24 h to detect PD-L1 and α-SMA levels. GAPDH is a loading control.

Supplementary Figure 3

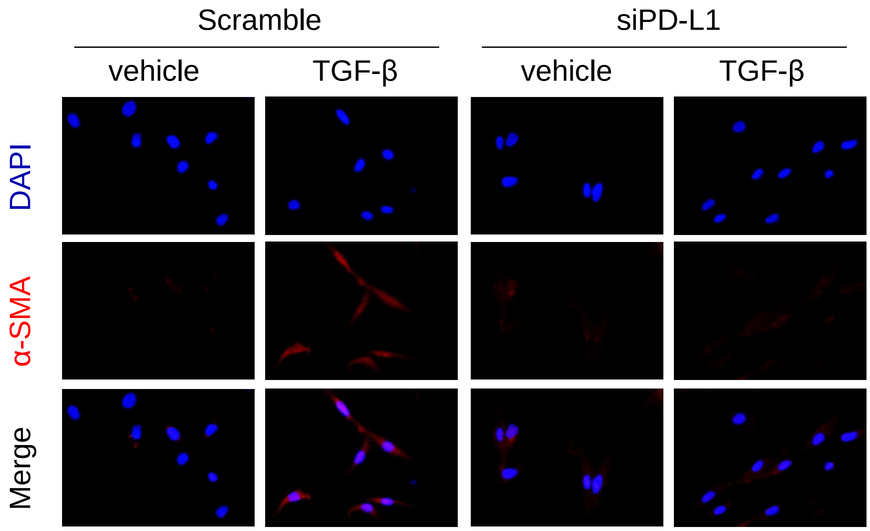

PD-L1 knockdown suppressed α-SMA localisation/presence induced by TGF-β as shown by immunofluorescence (IF) staining. Primary normal HLFs were transfected with scramble or siRNA targeting PD-L1 (siPD-L1), followed by treatment with TGF-β (5 ng/mL) for 16 h. Cells were then stained with α-SMA monoclonal antibody and detected with secondary immunofluorescence (IF) antibody. IF staining showed that knockdown of PD-L1 notably attenuated the induction of α-SMA (red) in primary normal HLFs. DAPI stained the nuclei.

Supplementary Figure 4

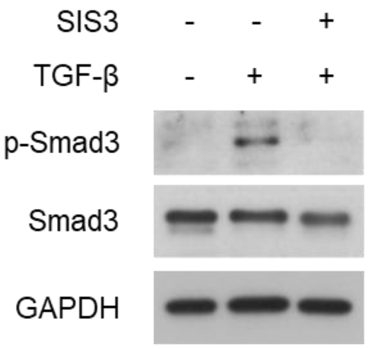

Primary normal HLFs were pretreated with SIS3 (10 μM) for 30 min, followed by TGF-β (5 ng/mL) treatment for additional 24 h. Whole cell lysates were collected for Western blotting analysis of phosphor-Smad3 (p-Smad3) and total Smad3 with GAPDH as a loading control.

Supplementary Figure 5

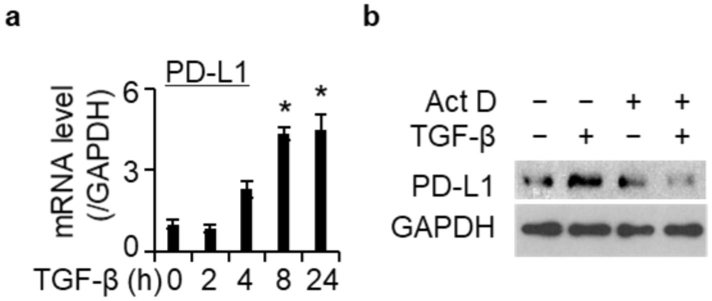

TGF-β increases PD-L1 at the transcriptional level in additional primary normal HLFs. **a**, qPCR data showed that TGF-β (5 ng/mL) induced PD-L1 mRNA expression in a time dependent manner in primary normal HLFs, which was significantly increased after 8 h treatment. n = 3 replicates. GAPDH is an internal control. **b**, Pretreatment with 1 μg/mL Actinomycin D (Act D) for 30 min blocked TGF-β (5 ng/mL) induced expression of PD-L1 in the primary normal HLFs. *, $P < 0.05$ vs. control group.

Supplementary Figure 6

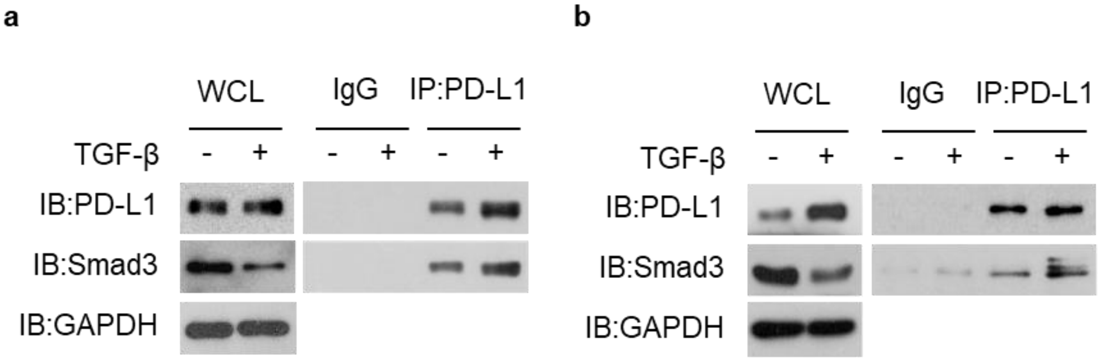

Co-IP showed an interaction between PD-L1 and Smad3 in IPF HLFs. **a** and **b** represent two independent experiments and were used for quantification in Figure 6c. WCL was collected from normal HLFs treated with vehicle or TGF-β (5 ng/mL) for 12 h, followed by incubation with IgG control or anti-PD-L1 antibody and immunoprecipitation as described in the methods.

Supplementary Figure 7

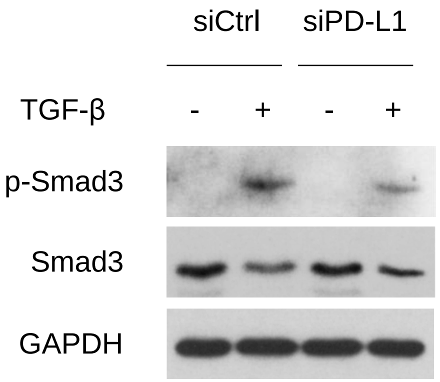

Primary normal HLFs were transfected with scramble (siCtrl) or siPD-L1, followed by treatment with TGF-β (5 ng/mL) for 24 h. The phosphorylation of Smad3, total Smad3 were detected by Western blotting. GAPDH is a loading control.

Supplementary Figure 8

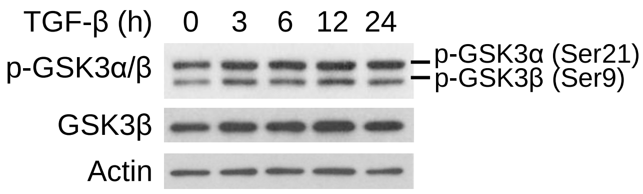

TGF-β induces inhibitory phosphorylation of GK3β (p-GSK3β, Ser9) in primary normal HLFs. The increase of p-GSK3β was observed as early as 3 h post TGF-β (5 ng/mL) treatment and prolonged up to 24 h, as examined.

Supplementary Figure 9

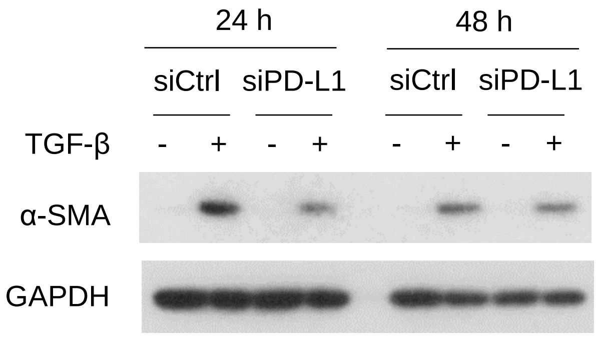

Primary normal HLFs were transfected with scramble (siCtrl) or siPD-L1, followed by TGF-β treatment (5 ng/mL) for different times (24 h and 48 h). The whole cell lysates were collected for Western blotting analysis of α-SMA. GAPDH is a loading control.

Supplementary Figure 10

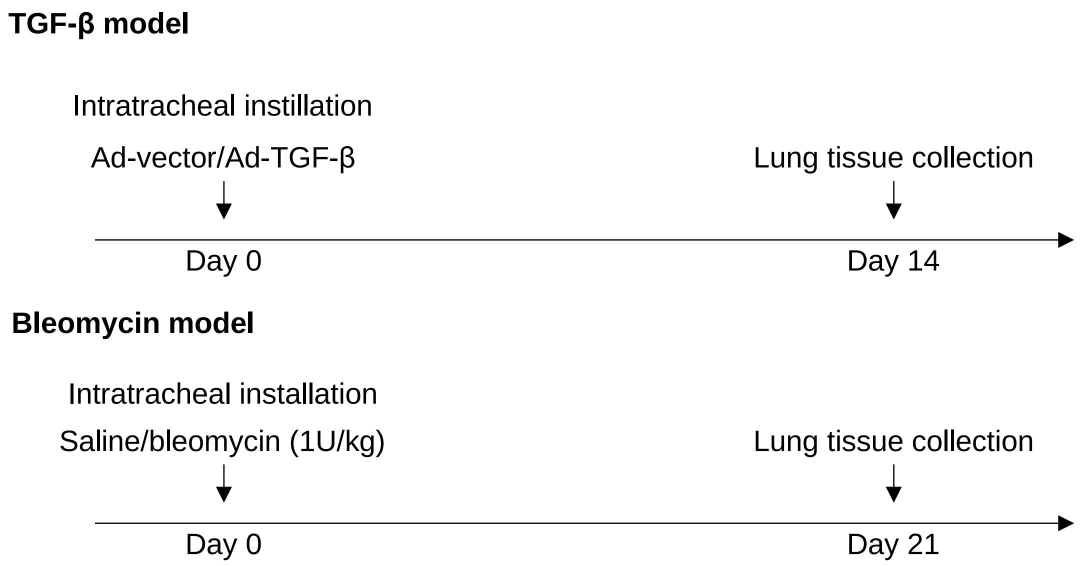

A diagrammatic view of the TGF-β and Bleomycin model used in the study.

Figure 3a: first experiment

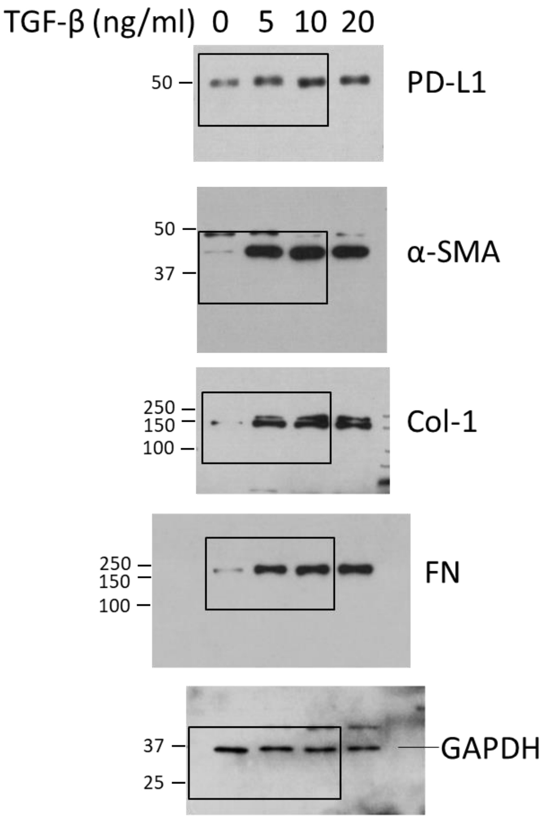

Figure 3a: second experiment

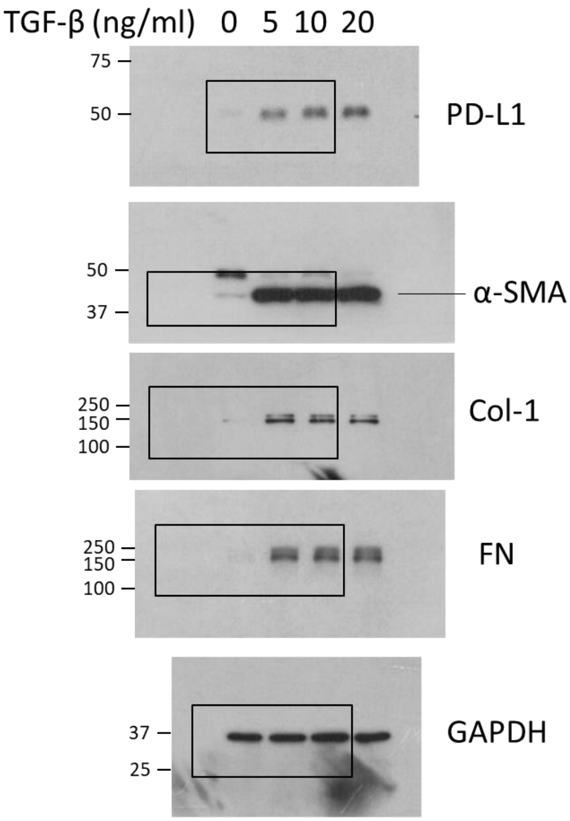

Figure 3a: third experiment

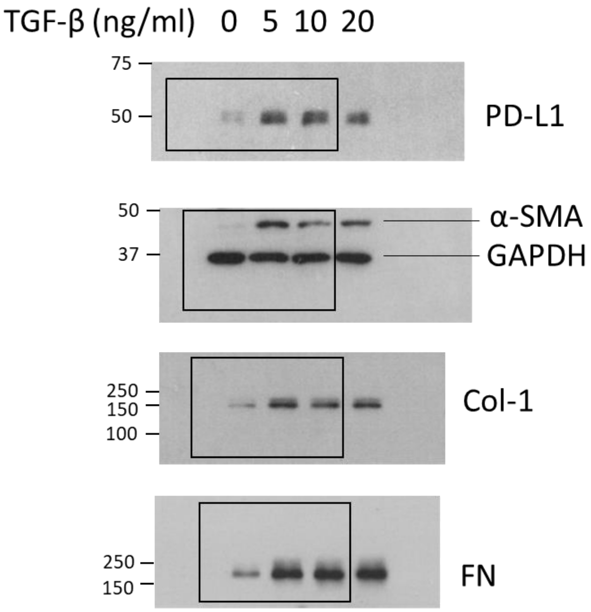

Figure 3c: first experiment

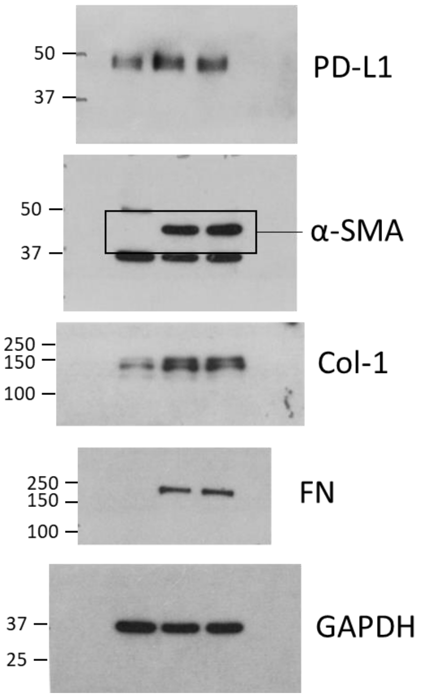

Figure 3c: second experiment

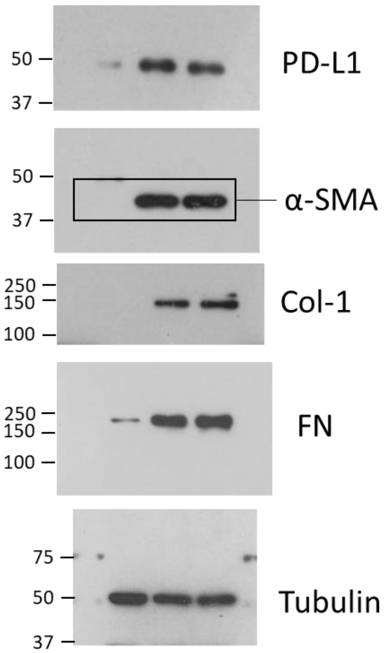

Figure 3c: third experiment

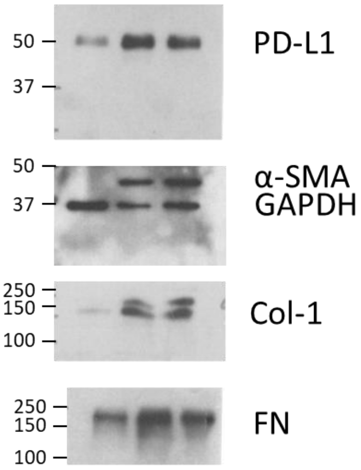

Figure 3e

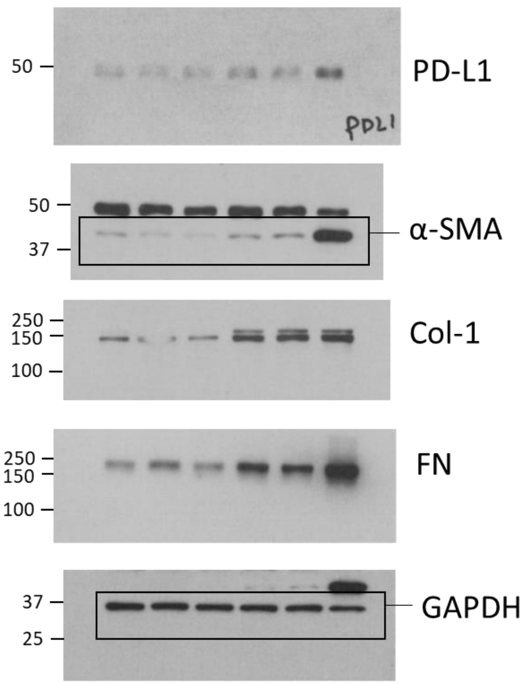

Figure 3f

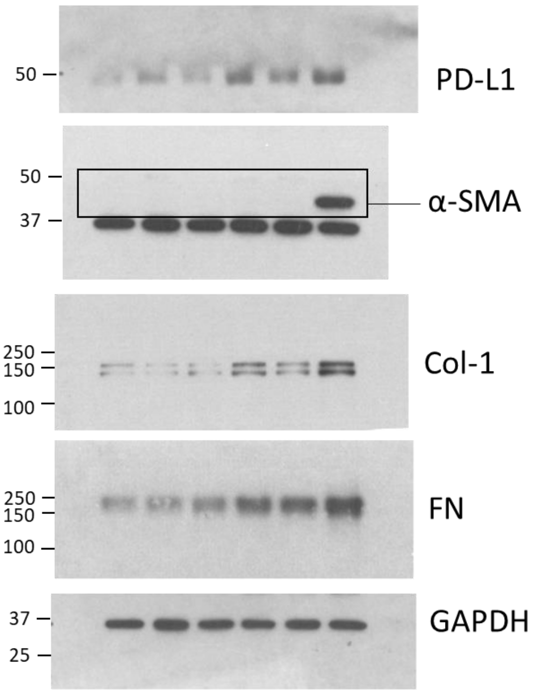

Figure 4a: first experiment

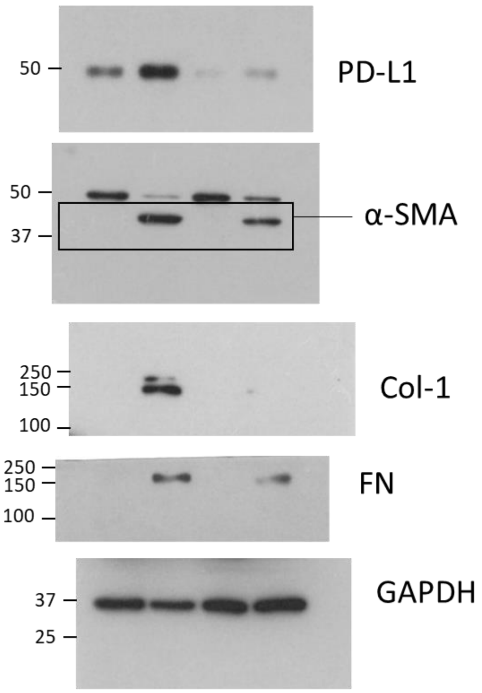

Figure 4a: second experiment

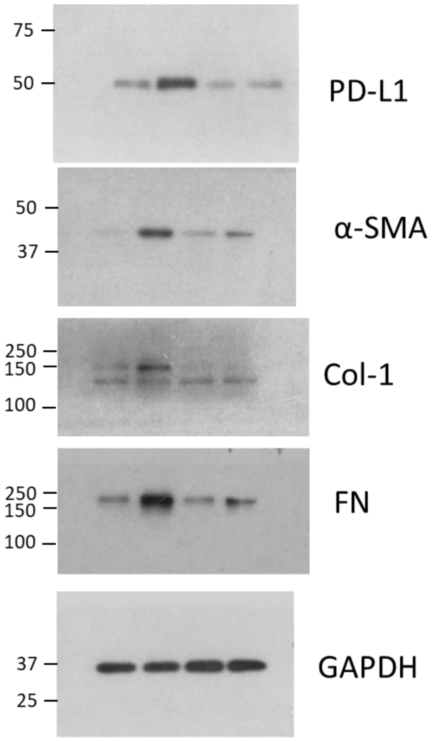

Figure 4a: third experiment

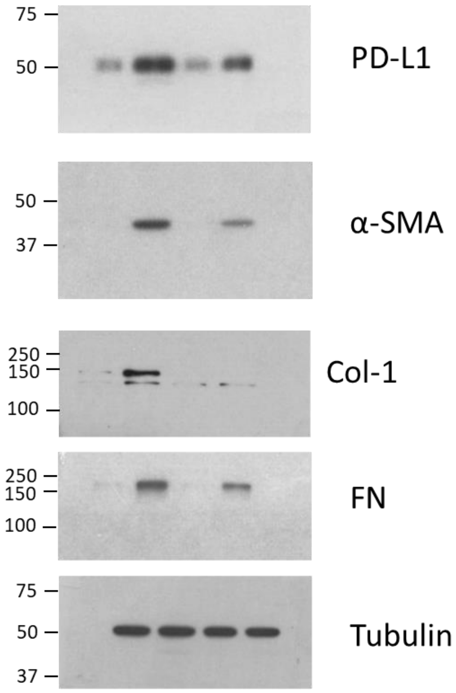

Figure 4c: first experiment

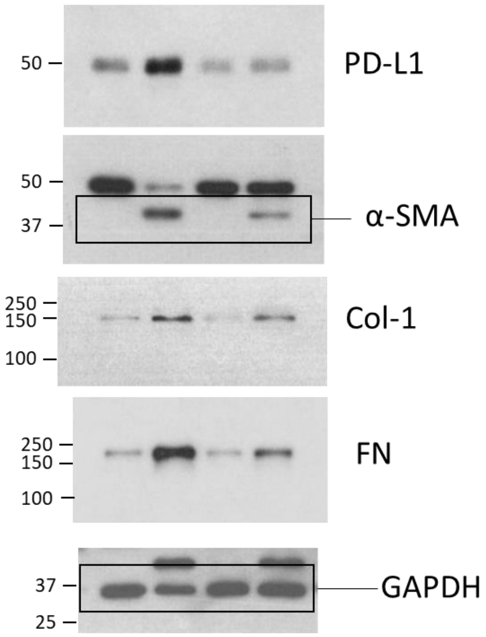

Figure 4c: second experiment

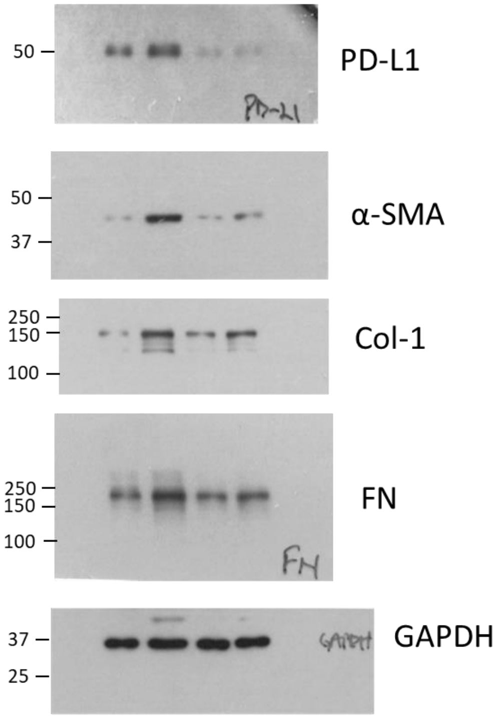

Figure 4c: third experiment

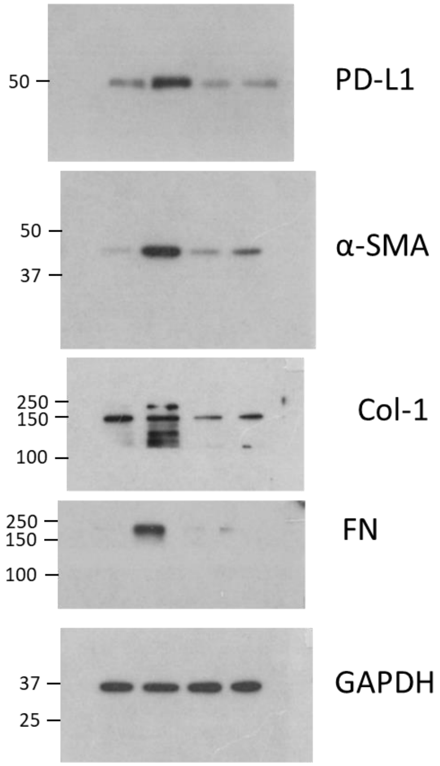

Figure 5a

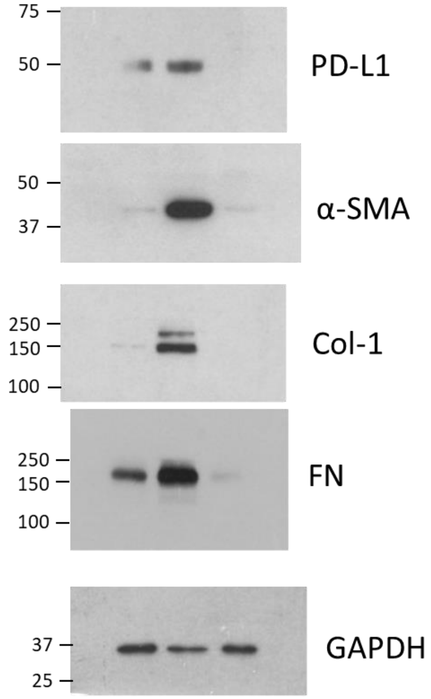

Figure 5b

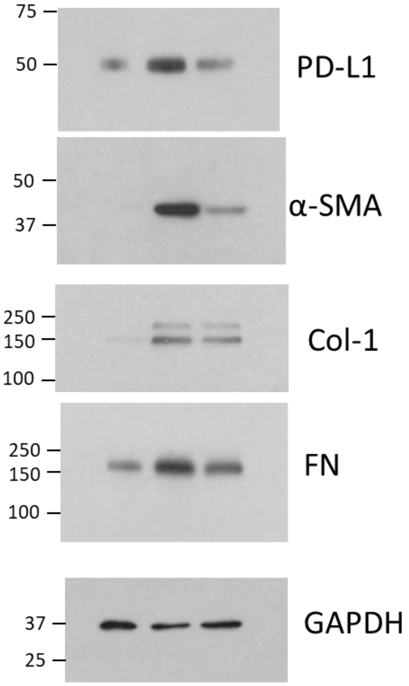

Figure 5e: first experiment

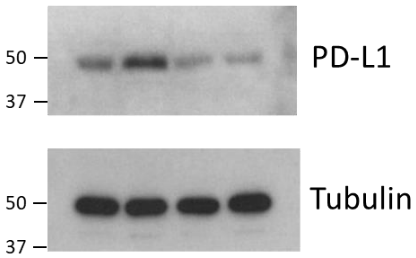

Figure 5e: third experiment

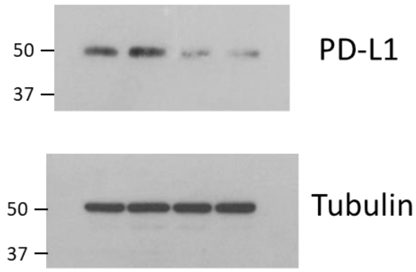

Figure 5e: second experiment

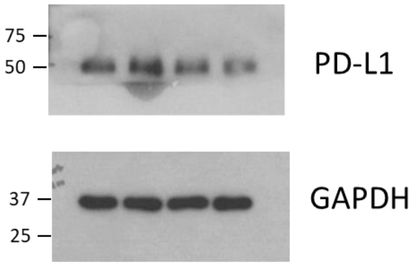

Figure 6b

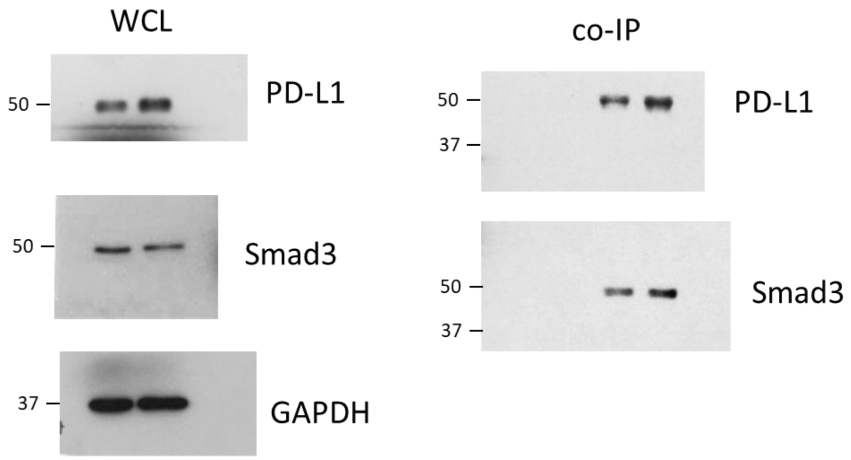

Figure 7a: first experiment

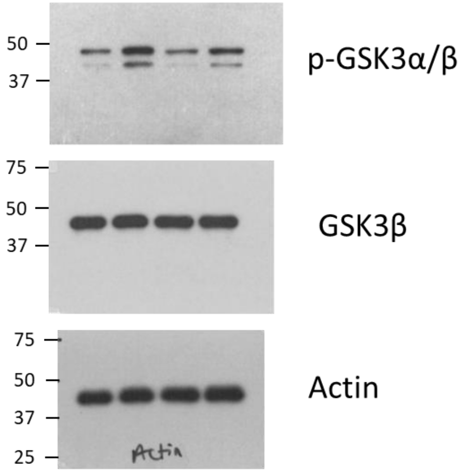

Figure 7a: second experiment

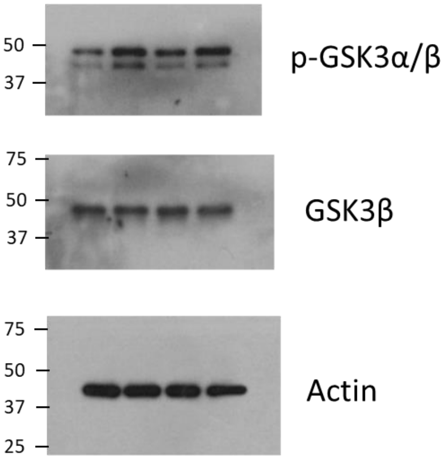

Figure 7a: third experiment

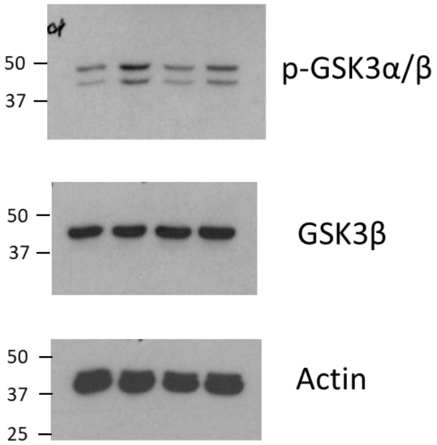

Figure 7c

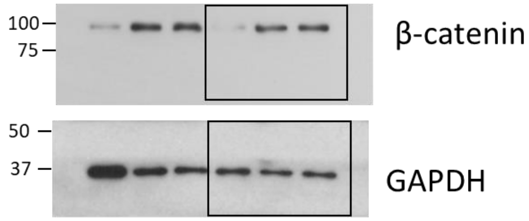

Figure 7d

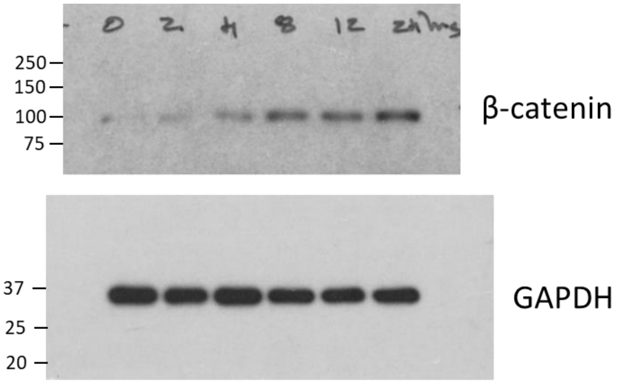

Figure 7e: first experiment

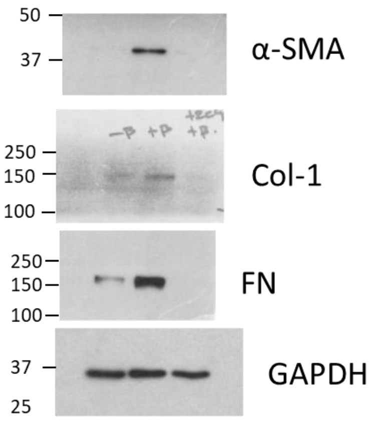

Figure 7e: second experiment

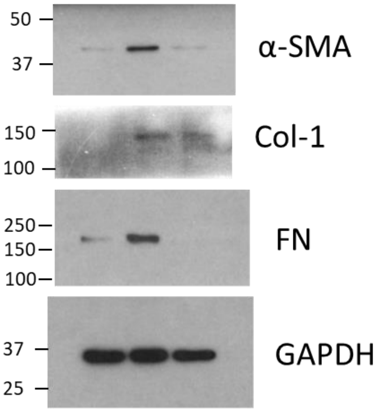

Figure 7e: third experiment

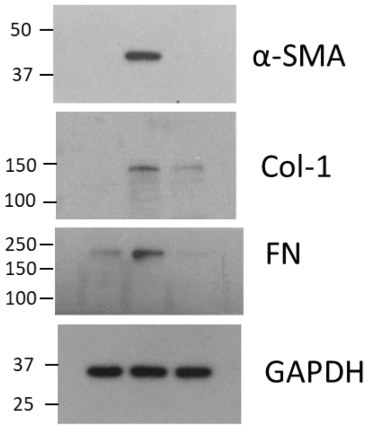

Figure 7g: first experiment

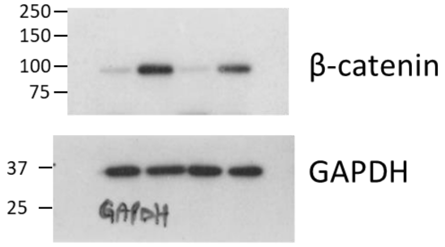

Figure 7g: second experiment

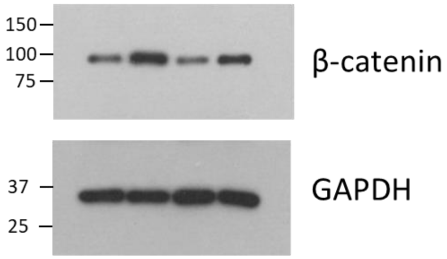

Figure 7g: third experiment

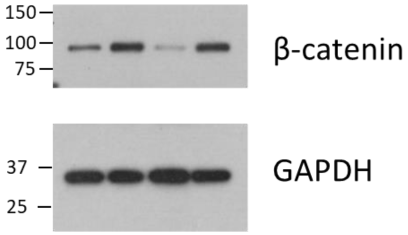

Supplementary Figure 2

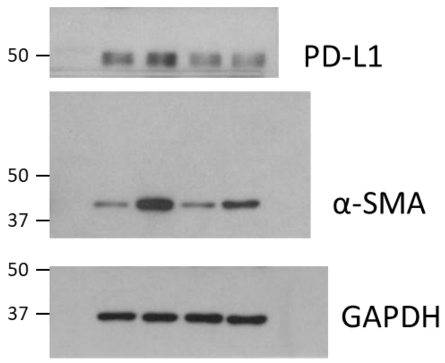

Supplementary Figure 4

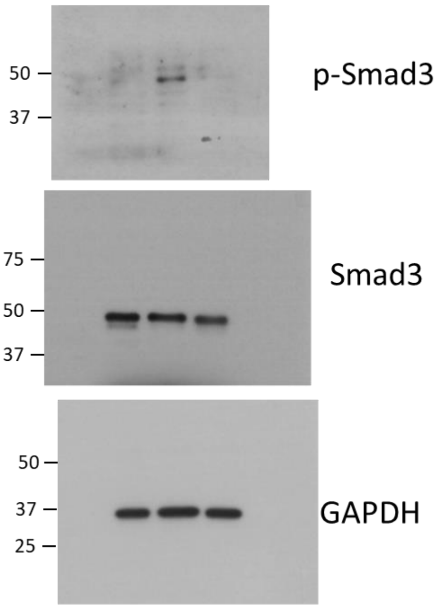

Supplementary Figure 5b

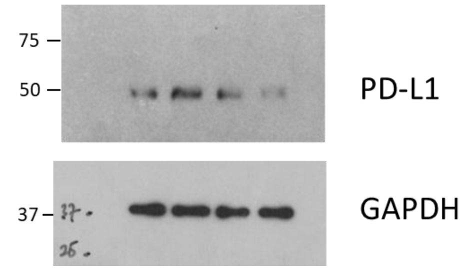

Supplementary Figure 6a

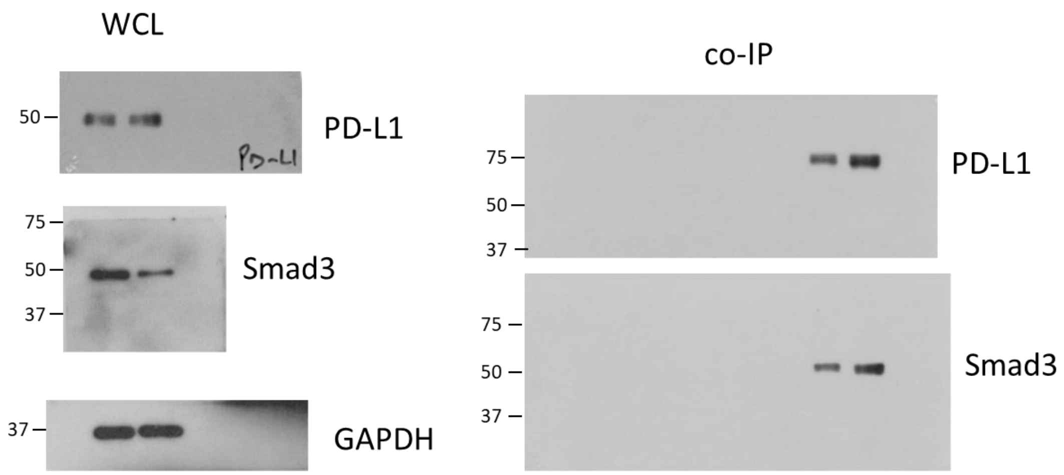

Supplementary Figure 6b

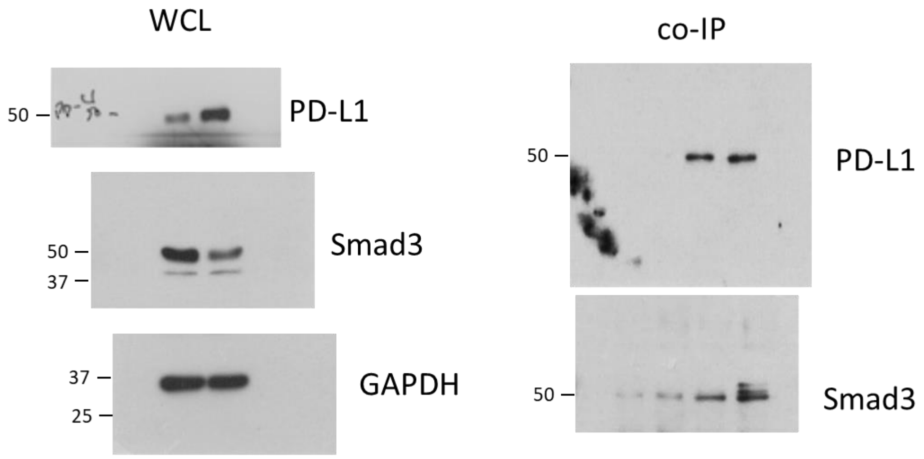

Supplementary Figure 7

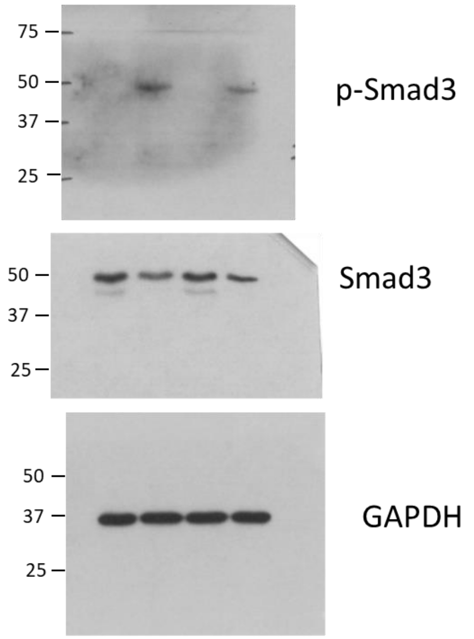

Supplementary Figure 8

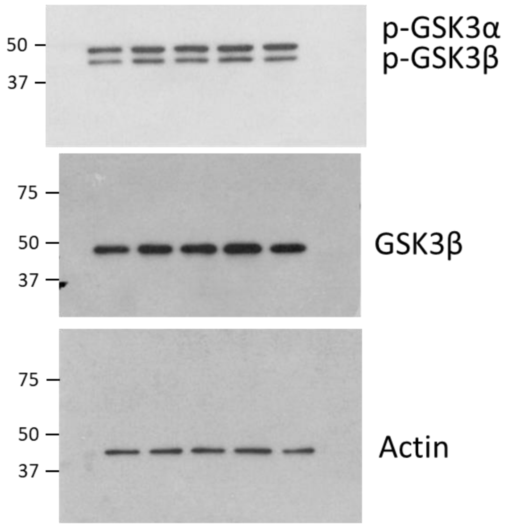

Supplementary Figure 9

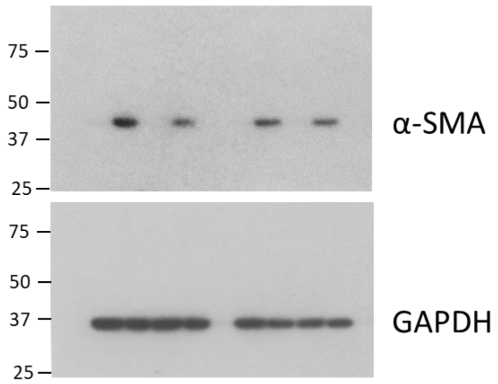

Supplement: Supplementary file 1 — Supplementary Information. [file 41598_2022_7044_MOESM1_ESM.pdf]
